# Composition engineered GNS/CNT Hybrid nanocomposites with enhanced thermal stability and tunable optical properties via one-step methane CVD

**DOI:** 10.1038/s41598-026-59863-3

**Published:** 2026-07-04

**Authors:** Mohamed M. Abdelhakim, Ahmed A. I. Khalil, Mostafa Alshershby, Abeer Salah

**Affiliations:** 1 Laser Sciences and Interactions Department, National Institute of Laser Enhanced Sciences (NILES), Cairo University, Giza, 12613 Egypt; 2 Laser and Optoelectronics Department, Technical Research Center (TRC), Ministry of Defence, Cairo, Egypt

**Keywords:** Graphene Nanosheets (GNS), Carbon Nanotubes (CNT), Hybrid Composites, Thermal Stability, Hybrid Nanostructure, Chemistry, Materials science, Nanoscience and technology

## Abstract

Graphene nanosheet/carbon nanotube (GNS/CNT) hybrid nanocomposites were produced using a one-step methane CVD method, employing a Mo–Mg–Fe catalytic system. This facilitated the concurrent growth and integration of graphitic elements. The impact of the Mo/Mg ratio on structural development, oxide chemistry, defect density, and thermal stability was examined in detail. SAED, TEM, and SEM revealed the coexistence of wrinkled graphene sheets linked with CNTs, while EDS and STEM showed even C, O, Mg, Mo, and Fe distribution. Raman indicated low defects (I_D_/I_G_ ≈ 0.14), I_2D_/I_G_ = 0.33, and A_2D_/A_G_ ≈ 0.43, which reveals that samples have common characteristics of few to multilayer graphene structures. FTIR reflected Mo/Mg-dependent oxide and surface chemistry, and UV–Vis revealed broad, enhanced optical absorption. TGA conducted in an inert atmosphere demonstrated remarkable enhanced thermal stability, characterized by negligible mass loss (2–6%) up to 1000 °C; this stability is ascribed to the formation of Mo-assisted carbides and the mitigation of Mg volatilization. The CVD method creates hybrids that are structurally low in defects. These hybrids show improved thermal stability and allow for the adjustment of their physical and chemical properties. As a result, these findings confirm that changing the catalyst’s composition is a useful way to design carbon nanohybrids with multiple functions.

## Introduction

Over the last two decades, carbon nanomaterials (CNMs), especially carbon nanotubes (CNTs) and graphene nanosheets (GNS), have attracted significant research interest due to their distinctive multifunctional attributes^1^. Understanding the synthesis processes and exploring their possible uses in new technologies has advanced significantly^2,3^. Their exceptional electrical properties, including near-ballistic transport and high carrier mobility, enable the creation of transparent conductive films, highly sensitive sensors, and sophisticated nano electronic devices.

Moreover, their remarkable mechanical properties—strength, stiffness, and flexibility—combined with their outstanding thermal conductivity, which facilitates efficient heat dissipation, make them ideal candidates for sophisticated composites and flexible or wearable electronic devices. In addition, the large surface area and easily modifiable surface chemistry of CNTs and GNS are widely exploited in energy storage, catalysis, and drug delivery applications, and their distinctive optical properties are increasingly used in photodetectors and biomedical imaging^4–6^. These materials could therefore prove useful in a wide range of applications, such as chemical sensors, photonics, solar cells, energy storage materials, catalytic supports, optical limiting, transistors, and water disinfection^7–25^. Researchers are combining these different forms of carbon to create hybrid structures. The combination of two-dimensional (GNS) and one-dimensional (CNT) or carbon nanofibers (CNF) to create three-dimensional hybrid materials expands the potential applications of nanomaterials. GNS/CNT hybrid materials can be made using various methods, including layer-by-layer self-assembly, electrodeposition^26,27^, liquid phase reaction^28,29^, and chemical vapor deposition (CVD)^30–38^. CVD is a widely used and effective method for making hybrids, offering excellent reproducibility, scalability, and control. This is done by growing carbon nanomaterials in one or two dimensions on different surfaces. 1D CNTs connect separate 2D graphene sheets, creating highly conductive pathways that greatly lower electrical resistance and make it easier to collect charge^39^. 1D materials interspersed between 2D layers act as physical spacers. This prevents the graphene sheets from restacking, which is a major issue in energy storage as it reduces the accessible surface area. Simultaneously, they reinforce the composite’s mechanical strength^40^. The junction between 1D and 2D materials creates unique interfaces where charge and energy transfer are optimized. This is critical for applications like photodetectors, where efficient separation of light-generated charges is needed^41,42^.

CVD proves to be more efficient than arc discharge and laser ablation in the synthesis of hybrid nanomaterials. CVD facilitates the control of parameters including temperature, pressure, and gas composition. This makes it possible to produce GNS-CNT on a large scale, with high purity and repeatability. More importantly, CVD allows for the direct growth of CNTs on graphene surfaces or from graphene-anchored catalysts, resulting in strong covalent or robust physical interfaces at the CNT-graphene junction, which is required for efficient charge transfer in sensing, energy storage, and optoelectronic devices. Furthermore, CVD runs under relatively moderate process settings as compared to arc-discharge techniques, providing better energy economy and compatibility with a wide range of substrates while preserving good structural and electrical quality in the resulting hybrids^43,44^. Zhuo et al. employed both MgO and Fe/MgO catalysts within a CVD method to synthesize graphene–carbon nanotube hybrid architectures^38^. Their findings revealed that the ratio of MgO to Fe/MgO in the catalyst was critical in determining the proportion of graphene to CNTs in the final material. Additionally, three-dimensional combinations of graphene and CNT architectures were fabricated using a CVD technique in two steps. Graphene was grown on a Cu foil, then CNTs were grown on top of it using an Fe/Al₂O₃/SiO₂/Si substrate. This two-step CVD method was also employed to grow 3D CNT/graphene hybrids^45^, and Lee et al. developed efficient CNT growth on reduced graphene oxide films^46^.

CVD has significant benefits over arc discharge and laser ablation for creating application-driven hybrid nanomaterials. In this configuration, Fe is the primary active site for hydrocarbon decomposition and carbon nucleation^47,48^, while Mo acts as a promoter, stabilizing Fe nanoparticles and preventing agglomeration^49^, and MgO provides a large surface area support, ensuring excellent dispersion and simple removal after growth^50^. The interaction of these components generates compact, stable catalytic nanoparticles that effectively decompose methane and facilitate uniform carbon nanotube formation, with metal ratios influencing yield and structure. Iron-based catalysts are widely used due to their ability to form carbides and scatter carbon^51–54^, while non-supported Fe, Co, and Ni oxides can efficiently catalyze graphene synthesis, with methane acting as a better reducing agent than hydrogen^55^. Fe is more cost-effective and environmentally friendly than Co or Ni^56,57^. Previous research has shown that GNS may be produced through dissociation of methane without porous Fe oxide catalysts as well as continuous graphene sheet synthesis utilizing unsupported Fe catalysts^58^. Both supported and non-supported Fe, Co, and Ni catalysts have been successfully employed for separate CVD synthesis of CNTs and GNS, and more recently, graphene nanosheets were synthesized on methane, which is used to produce nickel ferrite (NiFe₂O₄) at 800°C^59–61^. This highlights that one- and two-step CVD processes can produce GNS and their hybrid structures, making it relevant for the development of hybrid GNS–CNT–Mg–Mo–Fe nanocomposites with controlled morphology, interfacial quality, and enhanced properties.

As CVD can produce high-quality, low-defect structures with strong interfacial bonding, current literature strongly supports CVD as the ideal approach for manufacturing high-performance graphene-based and CNT–graphene hybrid materials. Unlike solution-based methods like Hummers’ method, which produce defect-prone graphene oxide (GO) or partially reduced rGO with poor conductivity, CVD enables the direct, one-step production of crystalline graphene and hybrids from gaseous precursors^62–65^.

Despite extensive studies on graphene and carbon nanotube nanostructures, the controlled formation of (GNS–CNT) hybrid architectures through a single-step (CVD) process remains a significant challenge. In particular, the influence of catalyst composition on the structural ordering and thermal stability of such hybrid carbon nanostructures has not been sufficiently explored. In this work, a one-step methane CVD approach is employed to synthesize GNS–CNT hybrid nanostructures with controlled Mo/Mg catalytic composition. The structural features and graphitic arrangement of the hybrid material are examined in detail through SEM, TEM, Raman spectroscopy, FTIR, and thermal analysis methods, including thermogravimetric analysis (TGA) and differential scanning calorimetry (DSC). The findings reveal that the Mo/Mg ratio plays a crucial role in determining the structural robustness and thermal resilience of the resultant hybrid carbon network. This provides novel perspectives on the design of thermally stable carbon nanostructures, which are essential for sophisticated photonic and thermal management applications.

## Materials and methods

This work used Sigma-Aldrich materials, including magnesium nitrate (Mg(NO₃)₂⋅6 H₂O), ammonium heptamolybdate (NH₄⋅Mo₂O₂₄⋅4 H₂O), and iron (III) nitrate (Fe(NO₃)₃⋅9 H₂O). Air Liquide provided the gases, including methane (CH₄, 99.995%), hydrogen (H₂), and nitrogen (N₂), which were used immediately without further purification.

### Preparation method of hybrid composites

CVD was used to synthesize the hybrid GNS-CNT-Mg-Mo-Fe nanocomposites. The MTI OTF-1200X configuration consisted of a horizontal quartz tube (100 cm length, 1.5 cm inner diameter) enclosed in an electrical furnace. Iron (III) nitrate, magnesium nitrate, and ammonium heptamolybdate were used as precursors for the preparation of the Fe–Mo–Mg catalyst. After calcination, the catalysts were placed in a quartz tube at the center of the furnace and employed for the methane CVD growth of GNS/CNT hybrid nanostructures. The furnace can reach 1200 °C. After stabilization, a gas mixture of methane (20 sccm) and hydrogen (50 sccm) was added for 30 min to initiate growth. The system was then cooled to room temperature under argon flow. Changing the catalyst’s Mo to Mg content ratio can have a substantial impact on the structure and morphology of the resulting carbon nanomaterials (CNMs)^37,55,66^. By changing this ratio, it’s possible to control how many GNS and CNTs are made in the hybrid structure. In this study, the synthesis was repeated using an optimized Mo/Mg ratio to validate and reproduce the selective growth behavior observed in earlier work. The findings confirm that Fe, Mo, and Mg catalysts give certain active sites that efficiently facilitate the GNS–CNT hybrid materials’ one-step growth.

### Catalyst preparation

Fe, Mo, and Mg catalysts contain 50% weight iron as the constant Fe content and weight ratios of 40/10, 30/20, 20/30, and 10/40 for Mo/Mg, in that order. Samples were synthesized using a straightforward liquid fusion method. Dissolve appropriate amounts of iron nitrate, ammonium heptamolybdate, and magnesium nitrate, each in 20 ml of distilled water, then mix them under sonication for 30 min to ensure homogeneity. The resulting solution was stirred at approximately 90 °C until the water evaporated and a uniform paste was formed. Following an overnight drying process at 120 °C, this paste was calcined for four hours at 500 °C. The solid that had been calcined was ground into a fine powder using an agate mortar, as shown in Fig. 2. The final catalyst, consisting of 50% Fe, 40% Mo, and 10% Mg by weight, was labeled as 40Mo-10Mg-Fe (S1). In the same way, other catalysts were made with varying Mo/Mg ratios but the same Fe concentration. These were labeled as 30Mo-20Mg-Fe (S2), 20Mo-30Mg-Fe (S3), and 10Mo-40Mg-Fe (S4), as summarized in Table 1.

**Table 1:** The prepared catalyst samples and their corresponding compositions.

Sample No.	Ratios
S1	40 Mo-10 Mg-Fe
S2	30 Mo-20 Mg-Fe
S3	20 Mo-30 Mg-Fe
S4	10 Mo-40 Mg-Fe

**Fig. 1:**
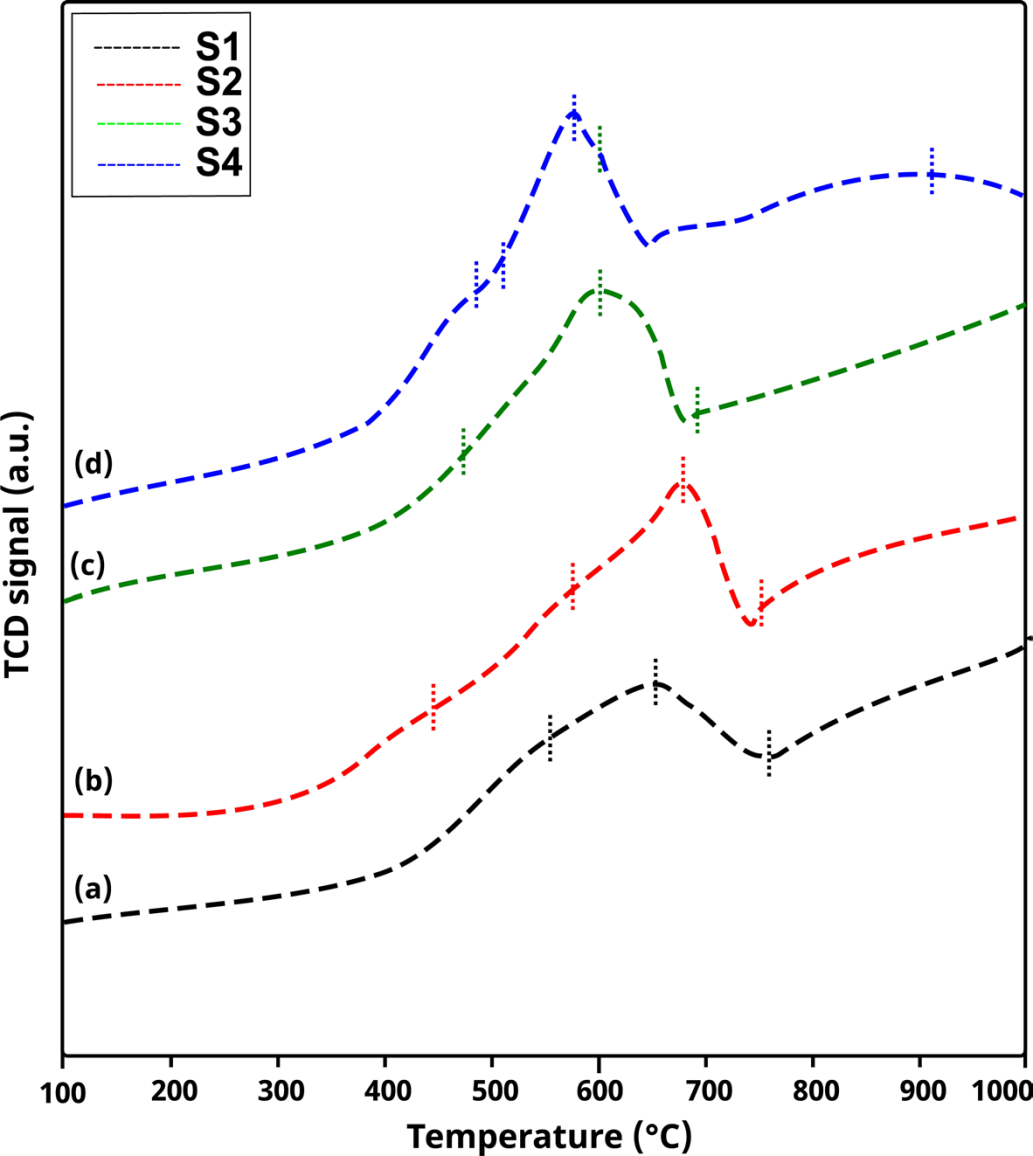
The profiles of H_2_-TPR for different Fe-Mo-Mg catalysts with different ratios of Mo/Mg (**a**) S1, (**b**) S2, (**c**) S3, and (**d**) S4.

The H_2_-TPR tests examined the impact of the Mo/Mg ratio on the reduction behavior of Fe-Mo-Mg catalysts. The TPR profiles indicated that the S1 catalyst displayed three broad reduction regions, highlighting a stepwise reduction process, with peaks at 542 °C and 647 °C linked to the co-reduction of hematite (Fe_2_O_3_) to magnetite (Fe_3_O_4_) and the reduction of MoO₃ to MoO_2_, along with partial reduction of FeMoO_x_ species^67–69^. A broad peak from 748 °C to above 1000 °C corresponded to the reduction of magnetite to metallic Fe and the hard reduction of tetrahedral Mo^6+^ to Mo^4+^. The presence of Mo was shown to hinder iron oxide reduction by forming Fe2(MoO4)3, suggesting substantial interaction between Mo and Fe oxide species. Increasing the MgO content to 20% in the S2 catalyst resulted in slightly higher reduction temperature peaks, attributed to the formation of mixed oxides like MgFe_2_O_4_ and MgMoO_4_, which enhanced Fe and Mo oxide dispersion but complicated the reducibility. As MgO content rose from 30% to 40%, reduction peaks shifted to lower temperatures, indicating easier reduction due to reduced Mo content. The S4 catalyst exhibited three peaks below 600 °C related to the reduction of Fe2O3 to FeO, while a broader peak above 650 °C was due to the reduction of FeO to metallic Fe and various MgFeO_x_ species, suggesting a significant amount of non-interacted bulk Fe_2_O_3_ as shown in Fig. 1. TPR results showed that the active phases of Fe-Mo-Mg catalysts were mainly iron molybdate and magnesioferrite species.

### The analysis of catalysts

The hybrid material of GNS and CNT was synthesized using a CVD process, with methane serving as the carbon source and Fe-Mo-Mg as catalysts. Approximately 0.5 g of catalyst was placed at the center of a quartz tube reactor (100 cm in length, 1.5 cm inner diameter) housed in an electrical furnace. To reduce the catalyst, the system was heated from ambient to 800 °C for an hour while pure hydrogen (60 sccm) flowed through it. After reduction, any remaining hydrogen was eliminated by purging the reactor with nitrogen (50 sccm). The GNS/CNT hybrid structures were then encouraged to grow by introducing methane (CH₄, 99.995%) during 2.5 h at a flow rate of 50 sccm. After the reaction, a constant nitrogen flow was used to cool the system to room temperature. The carbon nanomaterials were then collected for further examination and weighing.

**Fig. 2:**
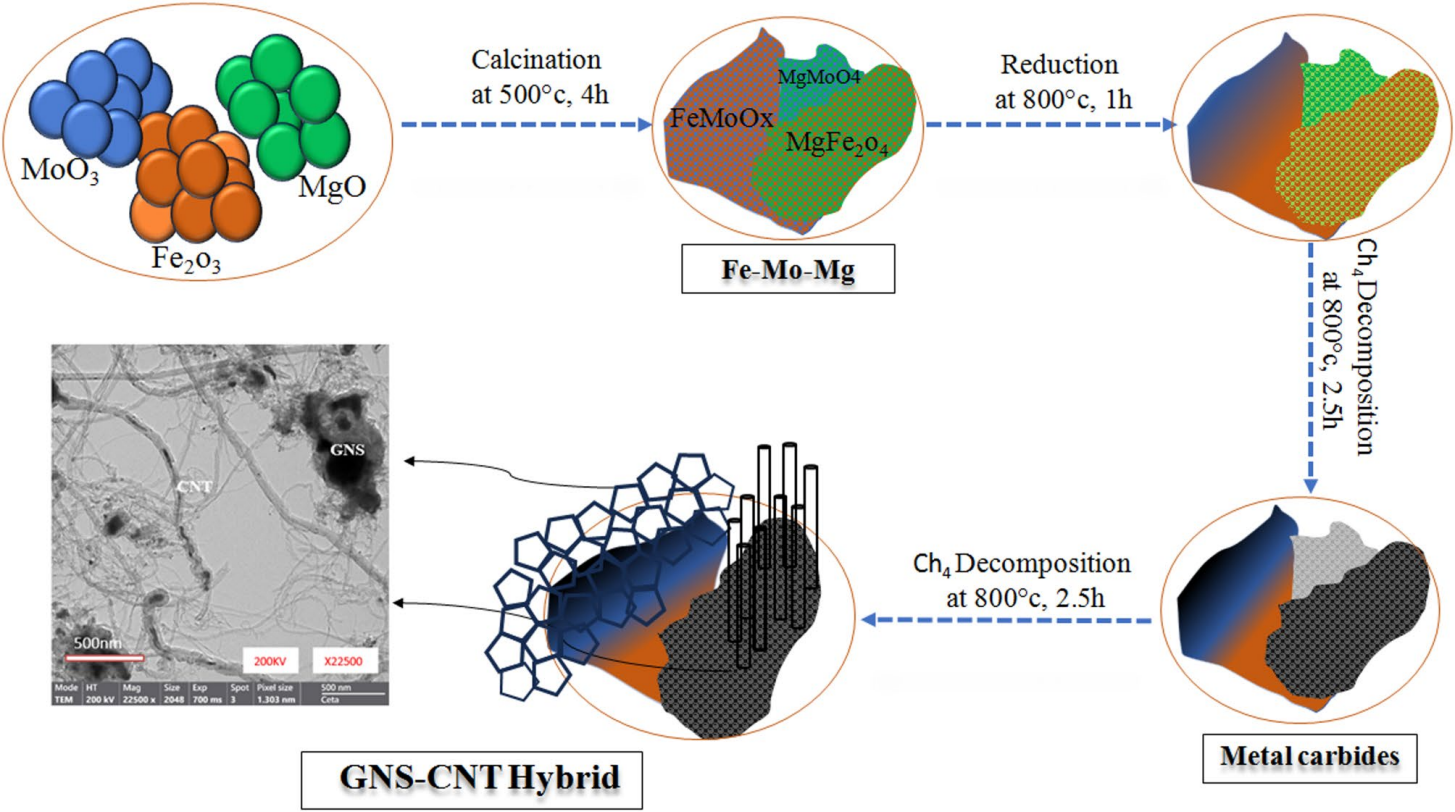
Schematic diagram illustrating the chemical vapor deposition (CVD) process used for synthesizing graphene nanosheet–carbon nanotube hybrid materials with Fe, Mo, and Mg catalysts.

### Hybrid GNS/CNT characterizations

The morphology and microstructure of the hybrid GNS–CNT samples were first investigated using high-resolution transmission electron microscopy (HR-TEM, JEOL TEM-2100, Japan) operated at 200 kV. To produce TEM samples, the hybrids were re-dispersed in 100% ethanol with an ultrasonic water bath. Before imaging, a drop of the suspension was set on a copper grid and left to dry. The surface morphology was further investigated using scanning electron microscopy (SEM), specifically a high-resolution field emission SEM (MIRA III XMU, TESCAN, Czech Republic). Both TEM and SEM provided detailed information about the nanomaterials’ hybrid structure. Elemental analysis was performed with an energy-dispersive X-ray spectrometer (EDS/EDX) coupled to the SEM. Raman spectroscopy was carried out on a Bruker Senterra II system (Germany) using a 532 nm Nd: YAG laser as the excitation source (10 mW power) and a Nikon 20x objective for focus. A PANalytical X’Pert PRO diffractometer (Netherlands) equipped with a CuKα radiation source (λ = 1.54060 Å) was used to measure X-ray diffraction (XRD). A 2θ range of 5.01°-89.99°, a step size of 0.02°, and a scan period of 0.5 s per step were used to collect the data. Measurements were taken at room temperature (25 °C) with a fixed divergence slit of 0.4785° and a receiving slit of 0.1 mm. To identify functional groups in hybrid materials, Fourier-transform infrared (FTIR) spectroscopy was performed using a Bruker Mobile IR-ATR spectrometer (Germany) in the 400–3500 cm⁻¹ range. Raman spectroscopy was carried out on a Bruker Senterra II system (Germany), with a 532 nm Nd: YAG laser as the excitation source (10 mW power) and a Nikon 20x objective for focus. Spectra were recorded in the range of 50–1800 cm⁻¹ to assess the structural quality and defect levels in the carbon network. Finally, optical absorption properties were evaluated using a JASCO V-770 High Performance UV/VIS/NIR spectrometer. Thermal stability was evaluated using a Mettler Toledo TA-TGA system. The samples were heated under a nitrogen atmosphere up to 1000 °C at a rate of 10 °C per minute.

### Results and discussion

The following formula was used to determine the yield of carbon nanomaterials (YCNMs)^70,71^:

1 $$\:{Y}_{CNMs}=\left(\frac{{m}_{final}-{m}_{catalyst}}{{m}_{catalyst}}\right)x100\:\:$$

**Fig. 3:**
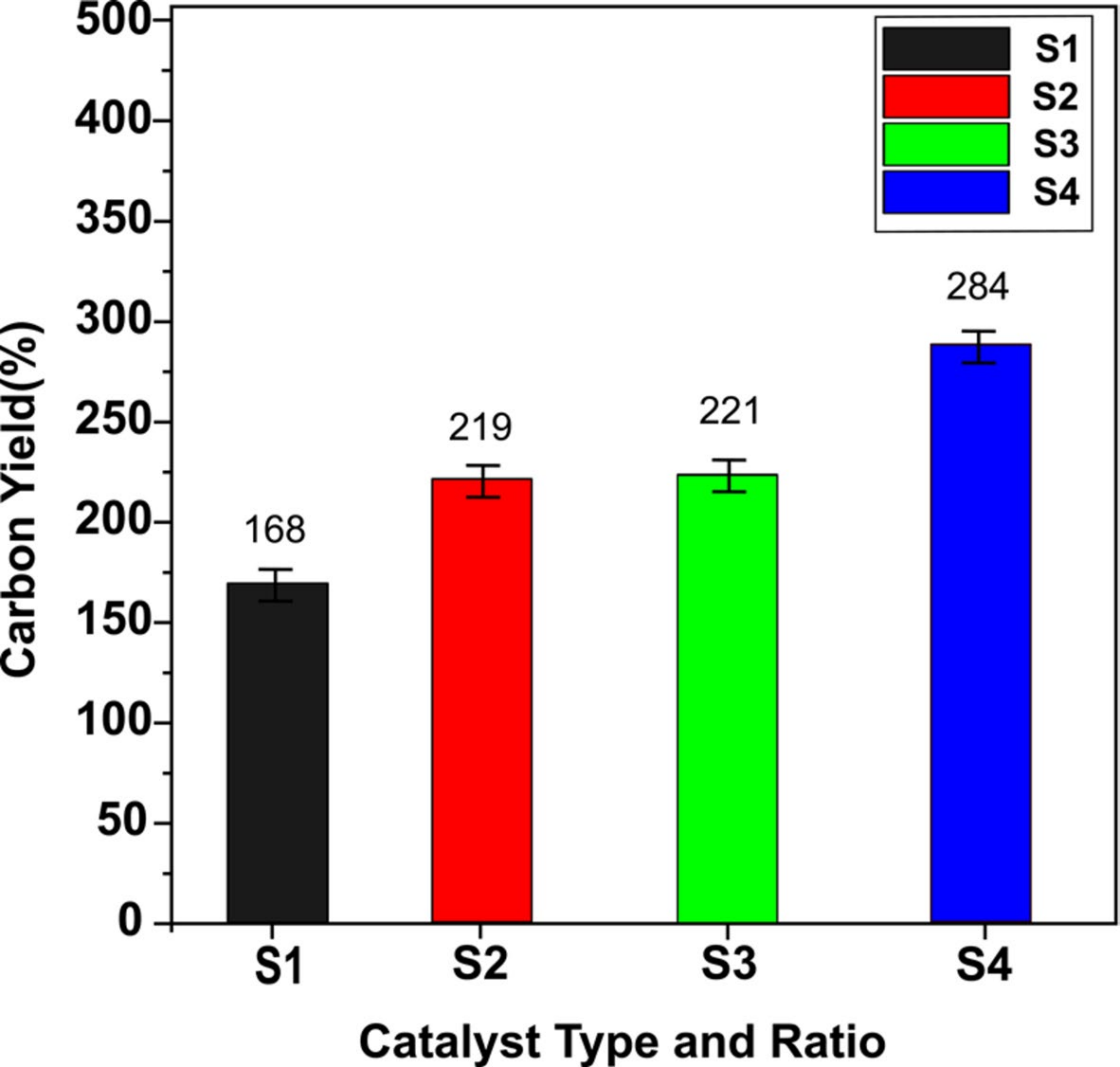
The total carbon yield from the decomposition of methane utilizing Mo-Mg-Fe catalysts with varying Mo/Mg ratios, including error bars.

In this equation, m_final_ represents the total mass after the reaction, which includes both the catalyst and the deposited carbon, while m_catalyst_ denotes the initial mass of the catalyst used. The resulting value, YCNMs, represents the yield of carbon nanomaterials as a percentage. Values obtained from multiple repeated experiments and black error bars added above each bar (± 6.5% for S1, ± 7.8% for S2, ± 8.2% for S3, and ± 9.5% for S4). As shown in Fig. 3, the amount of carbon deposited on the 40Mo-10Mg-Fe catalyst reached 168%. When the MgO content was increased to 20% and 30%, the carbon yield rose significantly to nearly identical values of 219% and 221%. This indicates that the active sites in these catalysts are very similar in both composition and behavior. When the MgO content was further increased to 40%, the carbon yield jumped to 286%, highlighting the strong influence of MgO on the catalytic performance. The current work is to use methane CVD across molybdenum, magnesium, and iron catalysts to produce hybrid nanostructured materials made of GNS and CNT. Therefore, it is crucial to determine the amount of carbon deposited after the CVD process is complete to evaluate the catalytic growth activities of Mo-Mg-Fe catalysts. It is shown that the CNM yield of Fe-Mo-Mg catalysts is influenced by their catalytic growth process, significantly impacted by varying the.

Mo/Mg ratio. Therefore, Fig. 3. shows the total amount of carbon made by different Mo/Mg ratios in Mo-Mg-Fe catalysts during 2.5 h of breaking down methane at 800 °C after multiple repeated experiments.

**Fig. 4:**
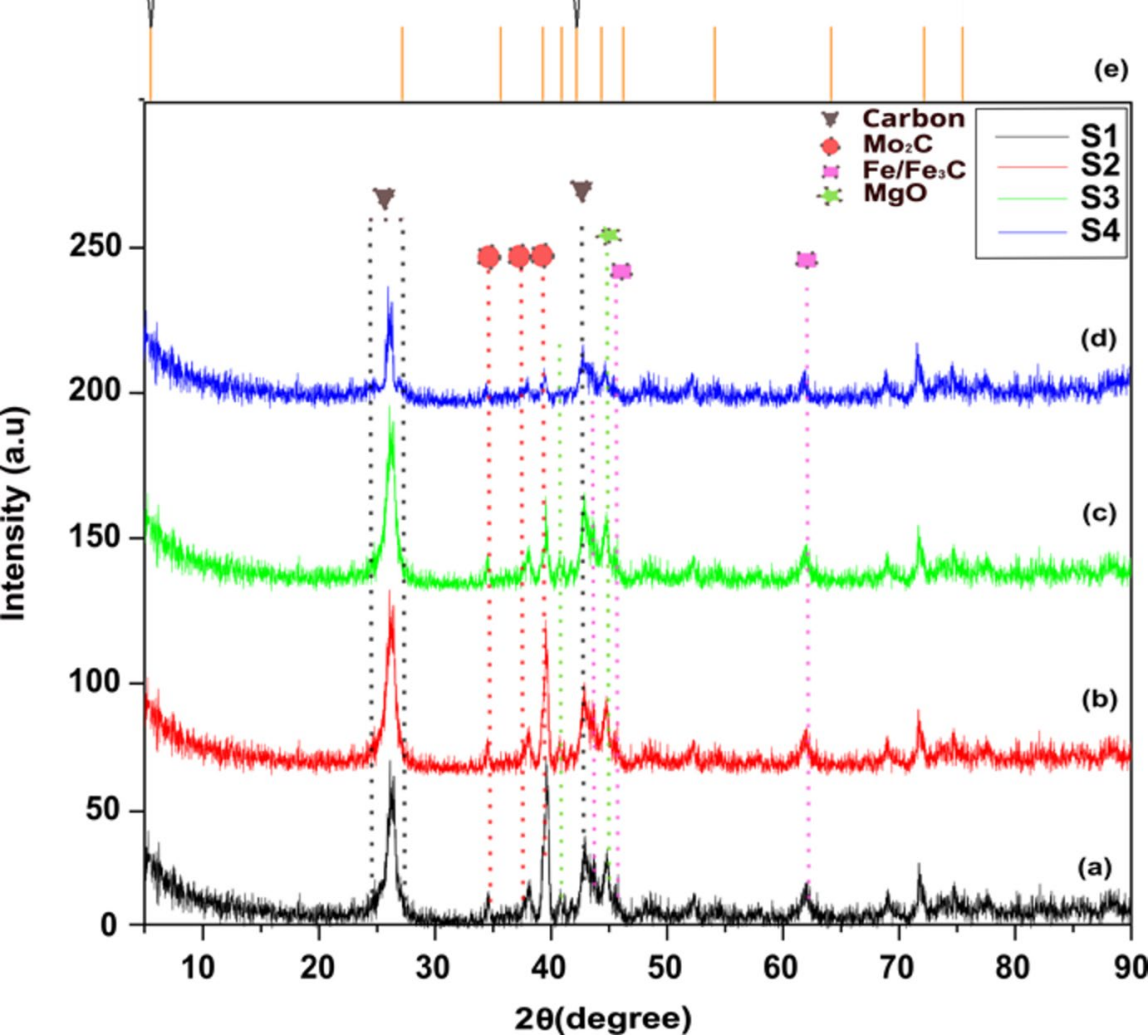
XRD patterns of Mo–Mg-Fe catalysts prepared with varying ratios of Mo/Mg: (**a**) S1, (**b**) S2, (**c**) S3, (**d**) S4, and (**e**) peaks agree with PDF/JCPDS.

According to the XRD results, the progressive rise in carbon output with a higher MgO content is mainly caused by the formation of new active sites such as MgFeO, alongside the existing FeMoOₓ and MgMoOₓ species, as shown in Fig. 1. Adding MgO also helps the catalytic metal particles disperse, which makes them more active overall. Adding MgO also helps disperse the Fe and Mo particles, which makes the CNT denser. This indicates that the number of active sites that make CNTs is directly related to the amount of carbon that is produced. As a result, the significant growth of GNS on the surface of the Fe-40Mo10Mg catalyst, as shown in Fig. 4(a), may explain its poor performance. Graphene sheets mostly cover the active sites, which makes it harder for methane to stick to them and break down. This lowers the amount of carbon that is released. This means that the type of carbon matters for how well the catalyst works. The carbon yield increases when the CNT yield also increases, not the GNS yield.

### XRD analysis

X-ray diffraction (XRD) was employed to examine the crystalline structures of GNS/CNT hybrid composites synthesized with Fe-Mo-MgO catalysts featuring varying Mo/MgO weight ratios, as depicted in Table 2. This comparison illustrates the impact of various catalyst compositions on the structural characteristics of the resultant hybrid materials. The XRD spectra of GNS-CNT-MgO-Mo-FeO hybrids exhibit a distinctive multi-phase crystalline structure. Graphitic carbon from the graphene and carbon nanotube network is present in all samples. A prominent diffraction peak centered at 26° indicates the (002) plane. The increased graphitic reflections at approximately 43° (100) further corroborate the formation of stacked graphitic layers. The presence of distinct diffraction peaks in crystalline MgO at 36.9° (111), 42.9° (200), 61.3° (220), and 74.7° (311) indicates effective cooperation among the MgO nanoparticles. As the concentration of MgO increases, the peaks intensify. The peaks at 34.4° (100), 37.9° (101), 39.6° (102), 52.1° (110), and 77.6° (211) indicate the formation of molybdenum carbide (Mo₂C) during chemical vapor deposition via carbothermal reduction. The reflections at 44.7° (110), 65.0° (200), and 78.0° (211) indicate the presence of iron and iron carbide phases (Fe/Fe₃C). The diffraction patterns of samples (a) and (d) indicate that the metal and oxide phases were more effectively integrated into the conductive GNS-CNT matrix. This indicates a general rise in crystallinity. The results indicate that a multi-phase hybrid structure made of graphitic carbon, MgO, Mo₂C, and Fe/Fe₃C was built successfully. This architecture has a lot of potential for energy storage, catalysis, EMI shielding, and composite applications that serve more than one purpose^14^. Metal carbides, especially those generated by Fe, Co, Ni, and Mo, have been shown in earlier studies to dramatically increase the formation of CNT and graphene during CVD^72–75^. The strong diffraction peak is located at 26.3984° (002) and has a height of 53.78 (100% relative intensity), as shown in Fig. 4.

The calculated d-spacing of 0.337 nm nearly aligns with the expected interlayer distance of graphite (~ 0.335 nm). The narrow FWHM value (0.2798° 2θ) indicates strong crystallinity and minimum structural disorder, indicating the presence of well-organized graphitic domains (GNS and CNTs)^76^. As shown in Table 2, these structural data confirm that the composite contains a highly crystalline graphitic phase. When MgO was introduced to the Fe-Mo catalyst alongside the hematite phase, weak diffraction peaks corresponding to MgFe₂O₃, MgMoO₃, and MgO formed, most likely due to interactions between MgO and Fe₂O₃/MoO₃ during calcination. MgMoO₃ results from a solid-state reaction between magnesium and molybdenum oxides.

**Table 2:** Data from XRD analysis of graphitic carbon deposited for GNS/CNT samples.

Pos. [2°Th.]	FWHM [2°Th.]	d-spacing [Å]	Relative Intensity [%]
26.39	0.2952	3.372	76.00
26.39	0.2798	3.376	100.00
26.49	0.3931	3.363	100.00
26.59	0.4712	3.345	100.00

Increasing the MgO content resulted in a marked reduction in diffraction peak intensities because FeMgOx, MgMoO₃, and MgFe₂O₃ are widely formed, suggesting stronger metal–support interactions and better spreading of Fe and Mo oxides^76,77^. MgO was used to modify the Fe-Mo catalyst to produce the hematite phase and weak diffraction peak for various substances, including Mn(OH)₂ + Mg(CO₃), which is associated with its high molecular weight. This was likely induced by interactions between MgO and FeO₃ or MoO₃. The existence of MgMoO₃ indicates that a solid-state interaction between molybdenum and magnesium oxides occurred.

### TEM analysis

**Fig. 5:**
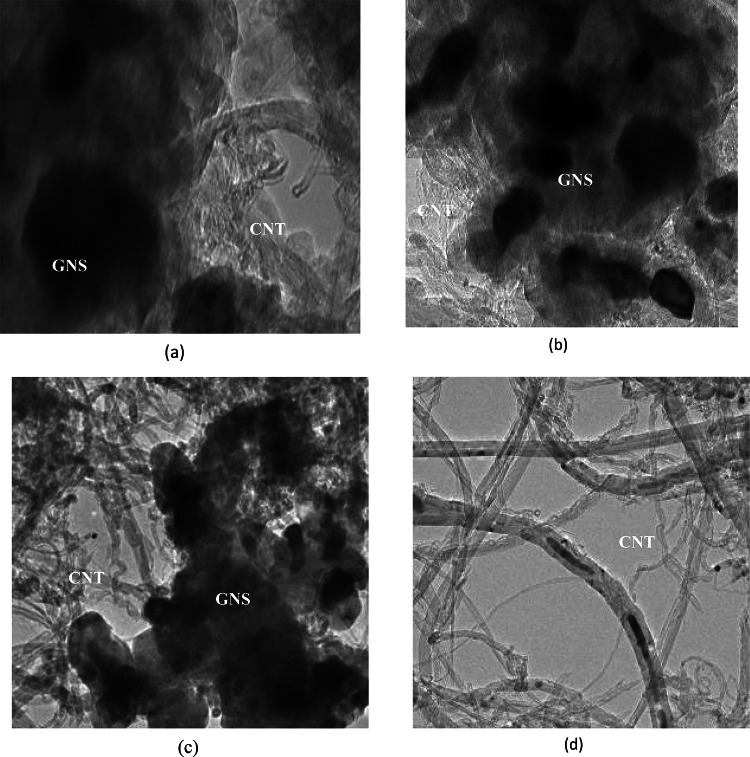

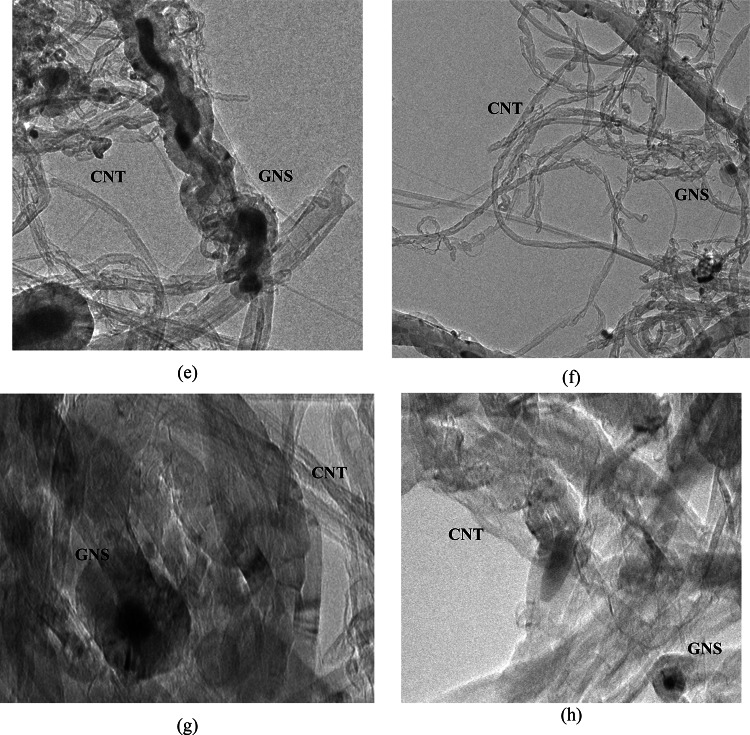

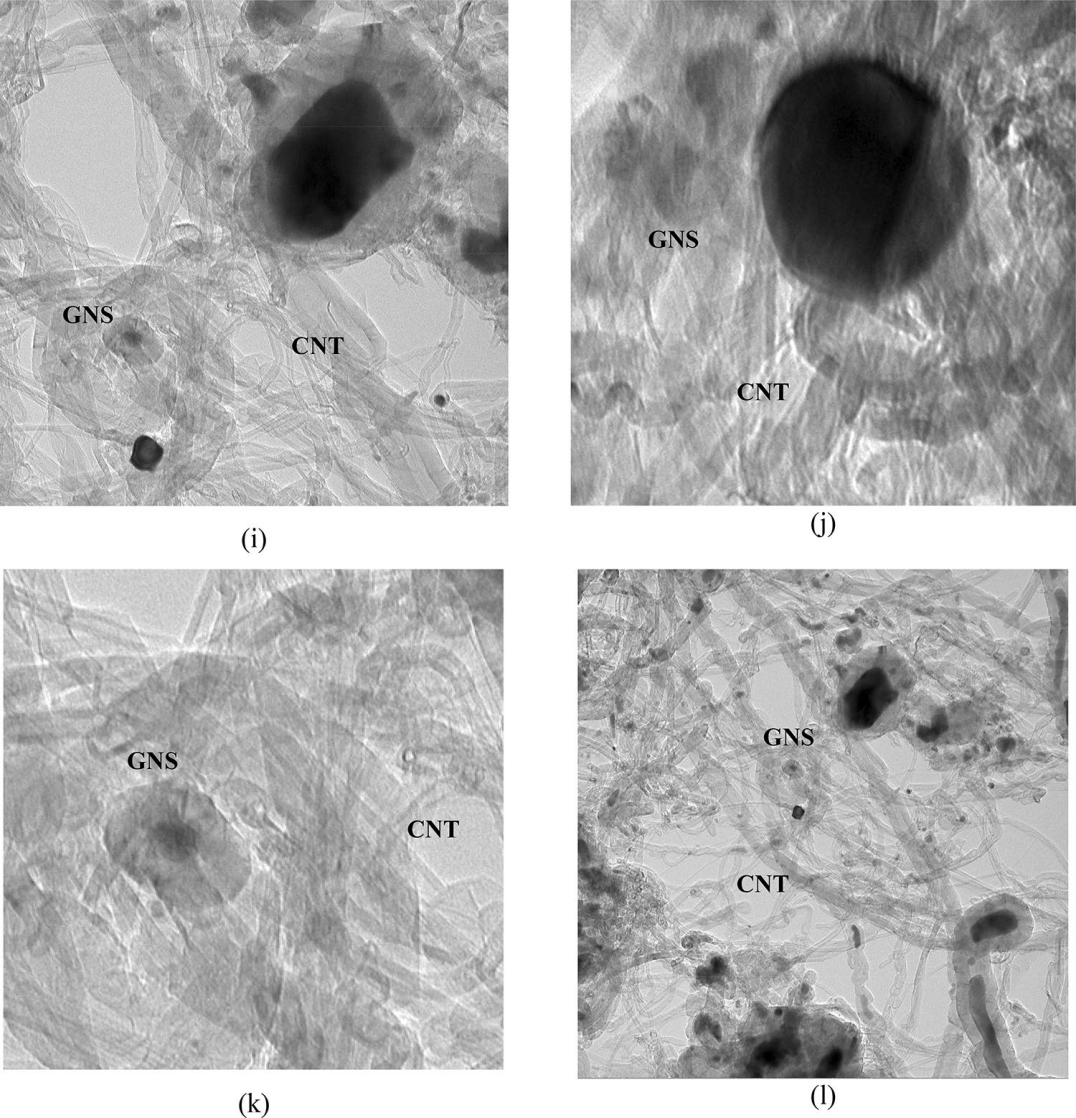

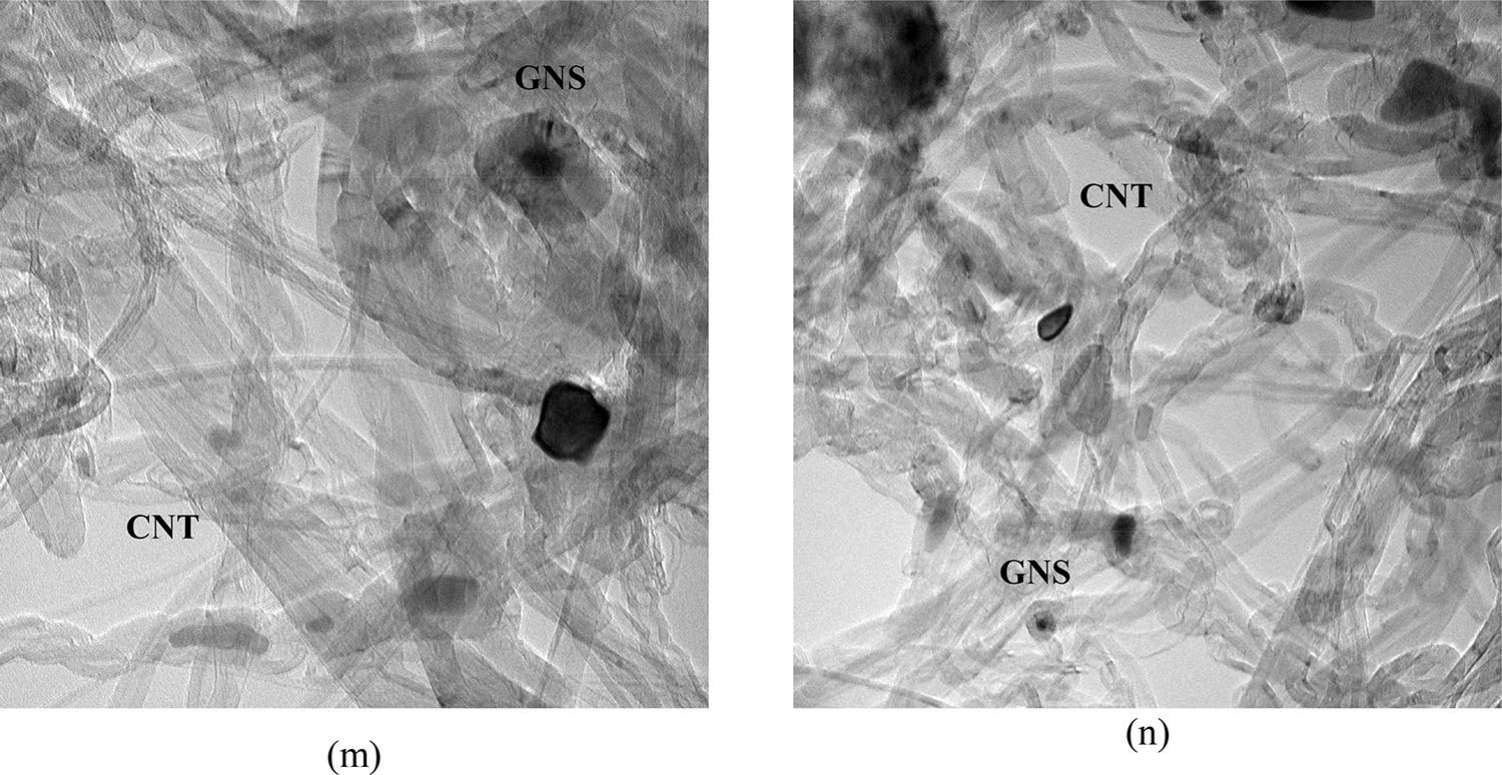
TEM and HRTEM images of hybrid GNS-CNT nanomaterials with different Mo/Mg ratios, (a-n) GNS-CNT, and (d) CNT.

TEM examination shows that the morphology of Fe-Mo-Mg catalyst nanoparticles employed for CVD growth of GNS and CNTs varies compositionally, as shown in Fig. 5. Table 3 summarizes the change from large, clustered particles at high Mo/low Mg (S1) to finely dispersed, uniformly distributed nanoparticles at higher Mg concentrations (S3 and S4) when the Fe content is fixed. This behavior highlights the role of MgO as an effective dispersant that suppresses sintering of the active Fe–Mo phases and increases the density of nanoscale catalytic sites. Well-dispersed Fe-based particles, particularly Fe₂O₃ interacting strongly with MgO, favor selective CNT growth, whereas aggregated FeMoOₓ species are mainly responsible for graphene nanosheet formation. Consequently, tuning the Mo/Mg ratio directly governs catalyst dispersion and phase distribution, enabling controlled adjustment of the relative GNS and CNT content in the resulting hybrid nanostructures. The catalyst morphology has a direct impact on CVD-grown carbon nanostructures, as nanoparticle size and dispersion govern CNT diameter, yield, and the layer continuity of GNS. The large, agglomerated particles in S1 serve as limited nucleation sites, potentially resulting in a low density of carbon nanostructures, which favors the production of few-layer graphene or thick CNTs via a base-growth mechanism.

**Table 3:** TEM Morphology Analysis Based on Mo/Mg Composition.

Sample	Mo (%)	Mg (%)	GNS/CNT Growth	Interpretation
**S1**	40	10	Forming few GNS and CNTs, or carbon shells on large particles; low yield due to low active sites; forming amorphous carbon.	Low Mg content leads to poor metal dispersion and a reduced number of active nucleation sites.
**S2**	30	20	Producing a mix of CNTs and GNS; moderate yield and best quality.	Mo promotes nucleation, while Fe supports subsequent carbon growth. Compared to S1, it provides more active nucleation sites for CNT/GNS growth.
**S3**	20	30	Increasing yield of thin CNTs and uniform GNS; best quality and structural control.	High Mg prevents sintering and maintains small active sites. Small, well-dispersed catalysts favor the tip growth of thin CNTs and uniform GNSs.
**S4**	10	40	Increasing ultrathin CNTs, few GNSs, and excessive encapsulation of particles by carbon.	High Mg dilutes active Fe-Mo sites, reducing CNT growth efficiency. Carbon can easily encapsulate tiny particles, suppressing continued growth.

S4 particles that are highly dispersed out can stick to carbon; this slows down GNS growth and enables CNTs to grow more. Among the compositions studied, S2 and S3 have more favorable morphology, combining small, well-isolated, and thermally stable nanoparticles that promote high-yield hybrid structures composed of thin CNTs interconnected with graphene nanosheets. According to literature, increasing Mo content promotes iron molybdate phase formation, with FeMoO₄ and Fe₂(MoO₄)₃ serving as active sites for GNS growth via methane decomposition. When reduced at 800 °C, these FeMoOₓ species transform into quasi-liquid Fe-Mo alloys that spread and form graphitic films^78,79^. High-resolution transmission electron microscopy (HRTEM) indicates images of the crystalline structure and carbonaceous materials. The central dark area shows well-defined atomic lattice fringes, indicating highly crystalline metal catalyst nanoparticles, mainly iron-molybdenum, originated from the CVD process. The first step in protecting graphitic shell formation is the enclosure of the nanoparticles by curved graphene layers^80,81^. CNTs take the form of hollow parallel structures that extend outwards. The majority of CNTs are multi-walled, featuring an inner core diameter of 0.3376 nm. The multi-layered, wrinkled sheets linking different tube networks distinguish the GNS background structures from the cylindrical CNTs. The morphological evaluations show that GNS occupy about 65% of the visible area and CNTs 35%. However, in denser zones around catalyst particles, the GNS/CNT ratio in other areas is about 55:45, indicating greater CNT concentrations correlating to larger catalyst particles. These regions are consistent with a balanced growth region in which CNTs uniformly dispersed over graphene substrates with little mass aggregation. The results indicate that the microstructure consists of approximately 58 vol% graphene nanosheets, a transparent multilayer support matrix, and 42 vol% interconnected mesh of multiwalled carbon nanotubes. The catalyst outer diameters obtained from the diameter measurements are in the range of 26–28 nm, while the CNT diameters are in the range of 8–14 nm (outer) and 3.5–5 nm (inner). GNS are seen to produce a lighter background with transparent crumpled sheets, and CNTs are identified as elongated intertwining filaments with connectivity in the material matrix. This detailed and varied magnification analysis reveals a consistent trend where GNS are present in wider fields, displaying a microstructure pattern that attests to the success of the one-step methane CVD process in creating a continuous three-dimensional material network^82^.

The addition of 10% MgO to the Fe–Mo catalyst facilitated the formation of hollow carbon nanotubes (CNTs) with diverse diameters, as illustrated in Fig. 5. Increasing the MgO concentration enhanced CNT yield by improving metal dispersion and generating MgFe₂O₃ and MgMoO₃ phases, thereby facilitating CNT synthesis. Previous studies have shown that MgMoO₃ nanoparticles enhance the formation of uniformly sized carbon nanotubes (CNTs) with varying Mo, Fe, and MgO ratios^83–89^.

### Selected area electron diffraction (SAED) analysis

The radial positions (interplanar spacings) and intensity distribution of the diffraction rings wrer examined to uniquely identify (GNS) and (CNTs) in these Selected Area Electron Diffraction (SAED) patterns. Both GNS and CNTs are allotropes of sp^2^-bonded carbon and as such they have the same core lattice properties. But the different geometries of the two (planar sheets and rolled cylinders) indicate different features in reciprocal space^90^. The simultaneous occurrence of continuous diffuse halos with sharply defined intense individual diffraction spots superimposed on the (002) and (100) rings confirms the presence of the combination of the curved multi-walled nanotube cylindrical walls with highly crystalline and flat planar domains typical of multi-layered graphene nanosheets, the corresponding SAED ring patterns of CNT bundles are given as insets, where the (002) graphitic planes were used to determine the experimental d-spacings indicating the co-existence of GNS and CNTs upon matching with the standard crystallographic values for graphite (sp^2^ carbon, hexagonal, space group: P6_3_/mmc) as shown in Table 4.^91,92^.

**Fig. 6:**
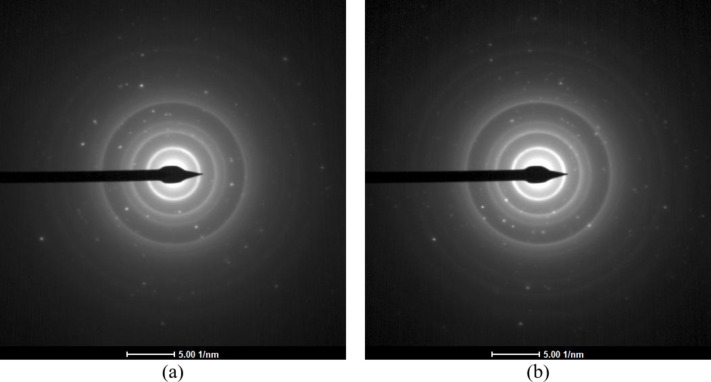
Selected area electron diffraction (SAED) pattern for different areas of the sample (**a**, **b**), The pattern from graphene is identified with white circles.

**Table 4:** The analysis of SAED images.

Ring Number (Innermost to Outermost)	Measured Radius (*R*)	Calculated d-spacing (d = 1/*R*)	Associated Miller Index (hkl)		Structural Proof Attribution (GNS vs. CNT)
Ring 1 (Brightest, Inner)	2.97 nm⁻¹	0.3376	(002)	Both represents the interlayer stacking distance.	
Ring 2	4.78 nm⁻¹	0.209	(100) / (101)	GNS-dominant in-plane honeycomb lattice.	
Ring 3	8.15 nm⁻¹	0.122	(110)	Both represent an atomic carbon-to-carbon framework.	

Carbon nanotubes are thin cylinders, and their curved geometry produces diffraction signatures that look like smooth, continuous, diffuse rings when sampled by an electron beam. The analysis also notes some particular observations in Fig. 6, remarking on the presence of a bright innermost ring, the (002) reflection at 0.3376 nm⁻¹, with weaker outer rings. The sharp, high-intensity individual dots on a continuous ring background are indicative of the presence of massive, highly crystalline, flat single-crystal domains, characteristic of laterally stretched, multi-layered graphene nanosheets. The innermost ring in the data is unusually strong and well-defined. It suggests the existence of multi-layered GNS (nanosheets) and multi-walled CNTs in the sample.

The rings are not of uniform intensity around the circle, with small arc-like variations. This is representative of a hybrid design where the GNS acts as a template or substrate, resulting in partial alignment or anchoring of the CNT network over the flat graphene surfaces during the CVD synthesis process.

### SEM and STEM (EDS/EDX) spectrum analysis

**Fig. 7:**
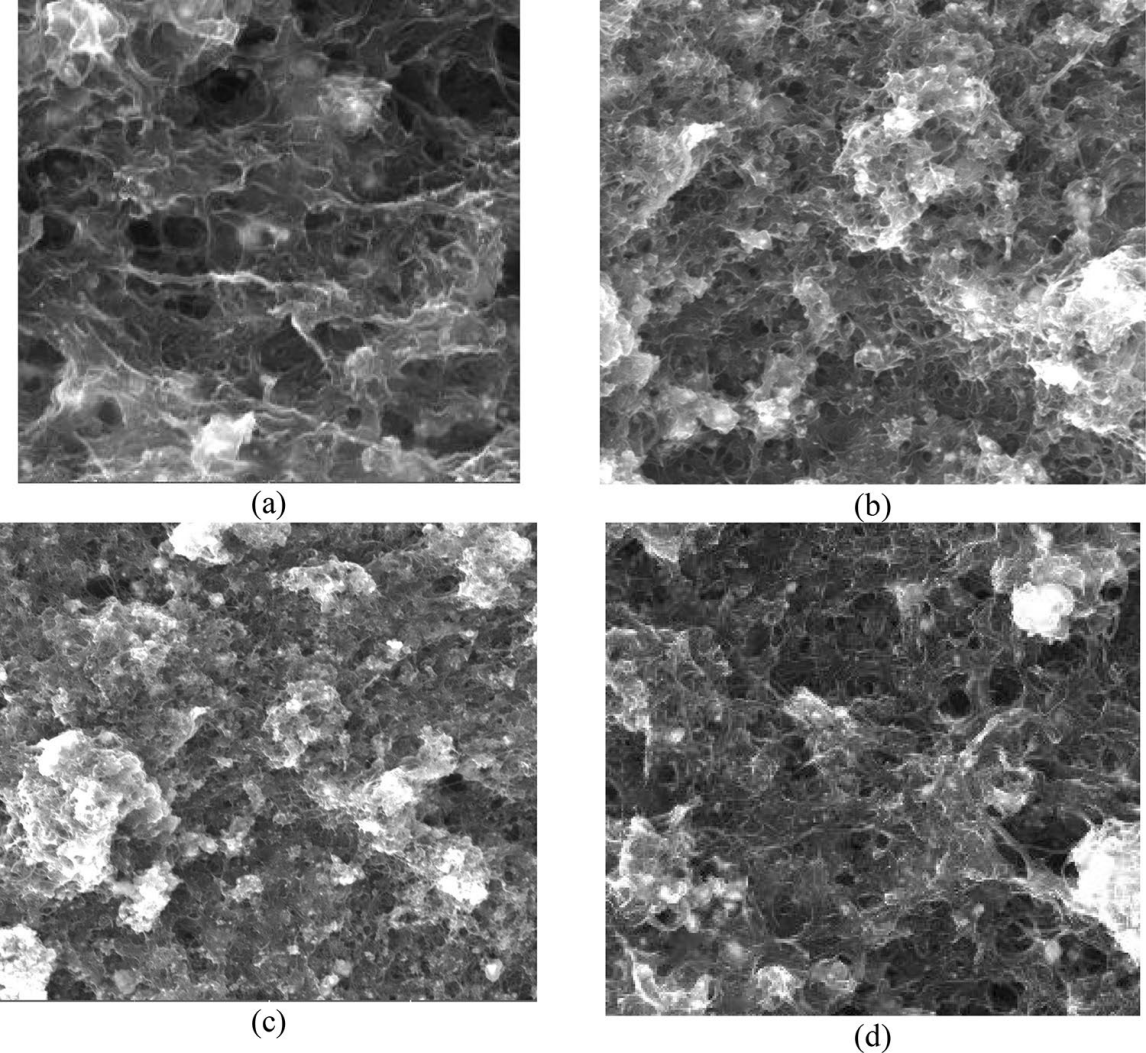

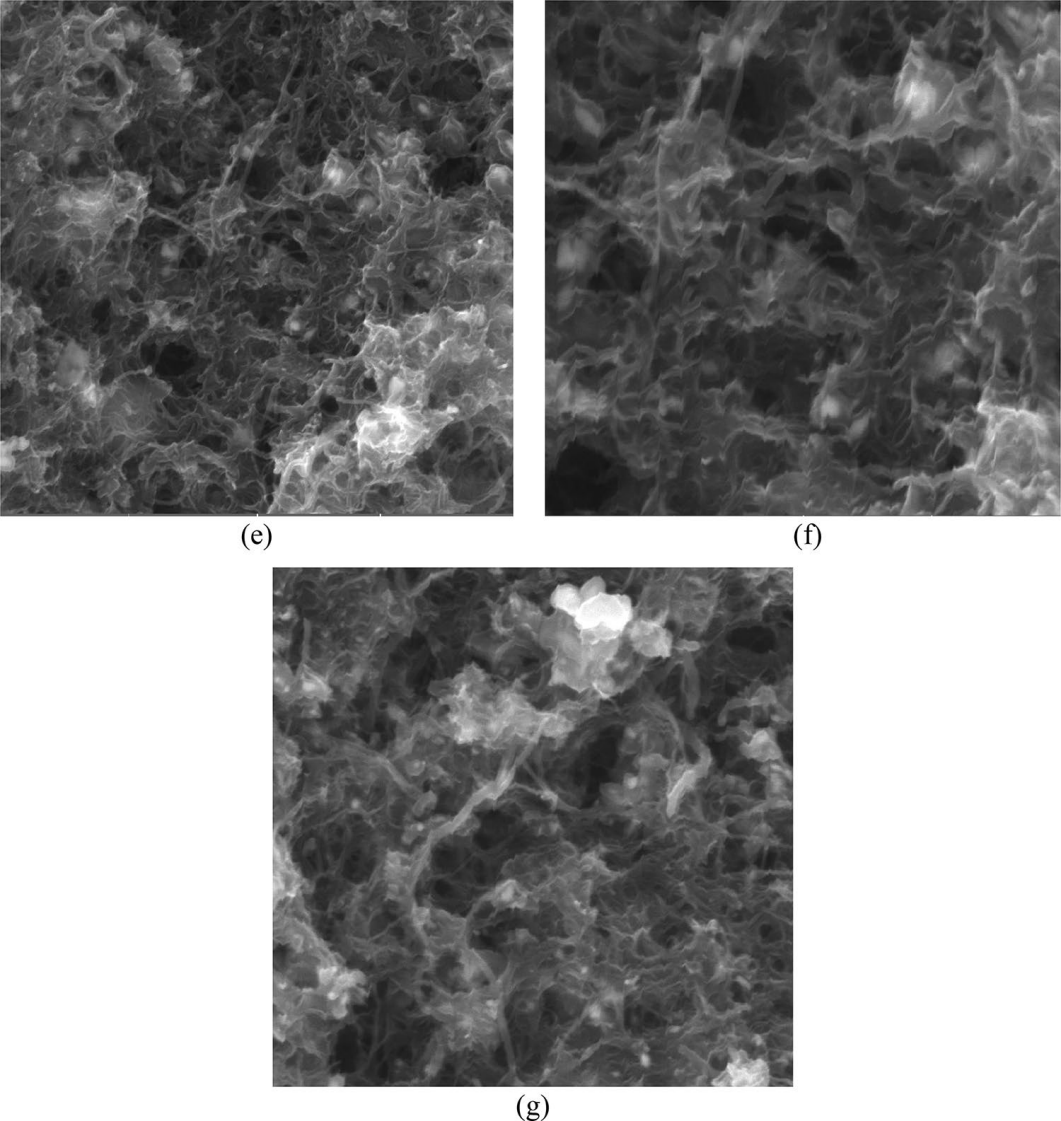
SEM images of hybrid nanomaterial (powder): (**a**) SEM for 1 μm (**b**) SEM for 5 μm, (**c**) SEM for 10 μm, and (a-g) SEM for 2 μm.

The SEM images of the hybrid nanomaterial have a highly processed and wrinkled look, indicating the presence of GNS rather than CNT, as seen in Fig. 7, due to their coarse texture. They appeared as thin, layered, interwoven structures that exhibit graphene’s folded and porous nature, which is compatible with its two-dimensional structure and stacking inclination. In contrast to CNTs, which are typically tubular, truncated, and lengthy. Due to its flat, sheet-like shape, graphene appears larger and darker in SEM images.

**Fig. 8:**
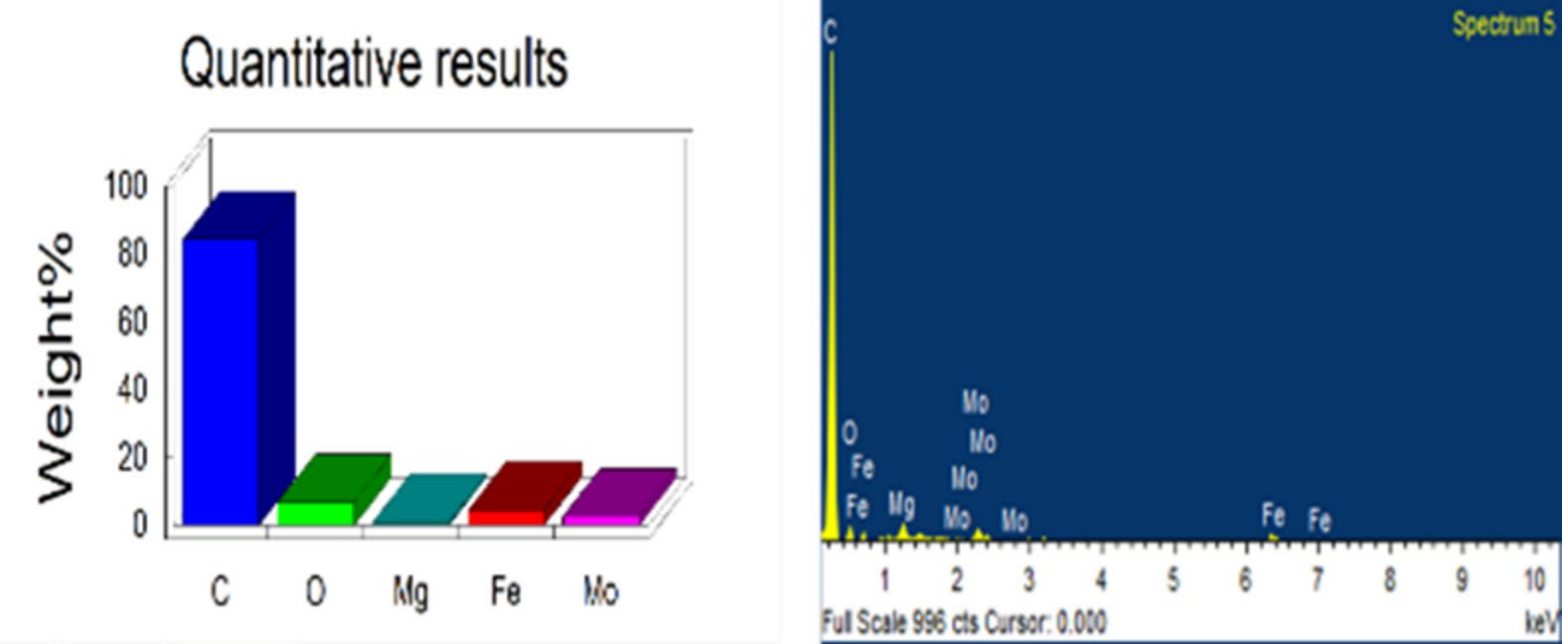
Energy Dispersive X-ray Spectroscopy (EDS/EDX) spectrum for samples, (**a**) Quantitative results, (**b**) Spectrum of EDS/EDX.

This morphology allows it to spread over a larger surface and absorb more electrons, resulting in lower brightness. Carbon nanotubes (CNTs), on the other hand, reflect more electrons than they absorb, giving an appearance of thinner, brighter strands. Heavy elements such as MgO, Mo, and Fe are responsible for the brighter areas of the hybrid structure’s SEM images, while carbon-based materials such as graphene and CNTs are seen in the darker regions^77,93,94^. This is further corroborated by SEM images which display two main structural components, namely a dense entangled CNT network and a supporting layer of crumpled GNS. The analysis also considers the micro-porous structure formed by the random orientation of CNTs on GNS, which avoids the restacking of graphene layers and enhances the interfacial contact during processing. Enhanced uniform dispersion of the material with no significant unreacted catalyst or bare graphene domains is evidence of effective synthesis. This visual difference helps in distinguishing between graphene and CNTs. Figure 8 shows the Energy Dispersive X-ray Spectroscopy (EDS/EDX) spectrum, which confirms the chemical composition of the hybrid composites for samples. A significant carbon (C) peak appears, indicating the presence of carbon-rich components such as graphene and CNTs. Oxygen (O) is also identified in MgO and potential oxide layers on Mo and Fe. The presence of magnesium (Mg) indicates the enhanced integration of MgO nanoparticles. Several different peaks for Mo and Fe indicate the presence of Mo-based compounds like MoO₃ and iron species like Fe₂O₃ or Fe nanoparticles. The dispersion of Mo and Fe across many peaks suggests that these elements are evenly distributed throughout the sample. The significant carbon signal shows the enhanced integration of carbon nanostructures.

**Fig. 9:**
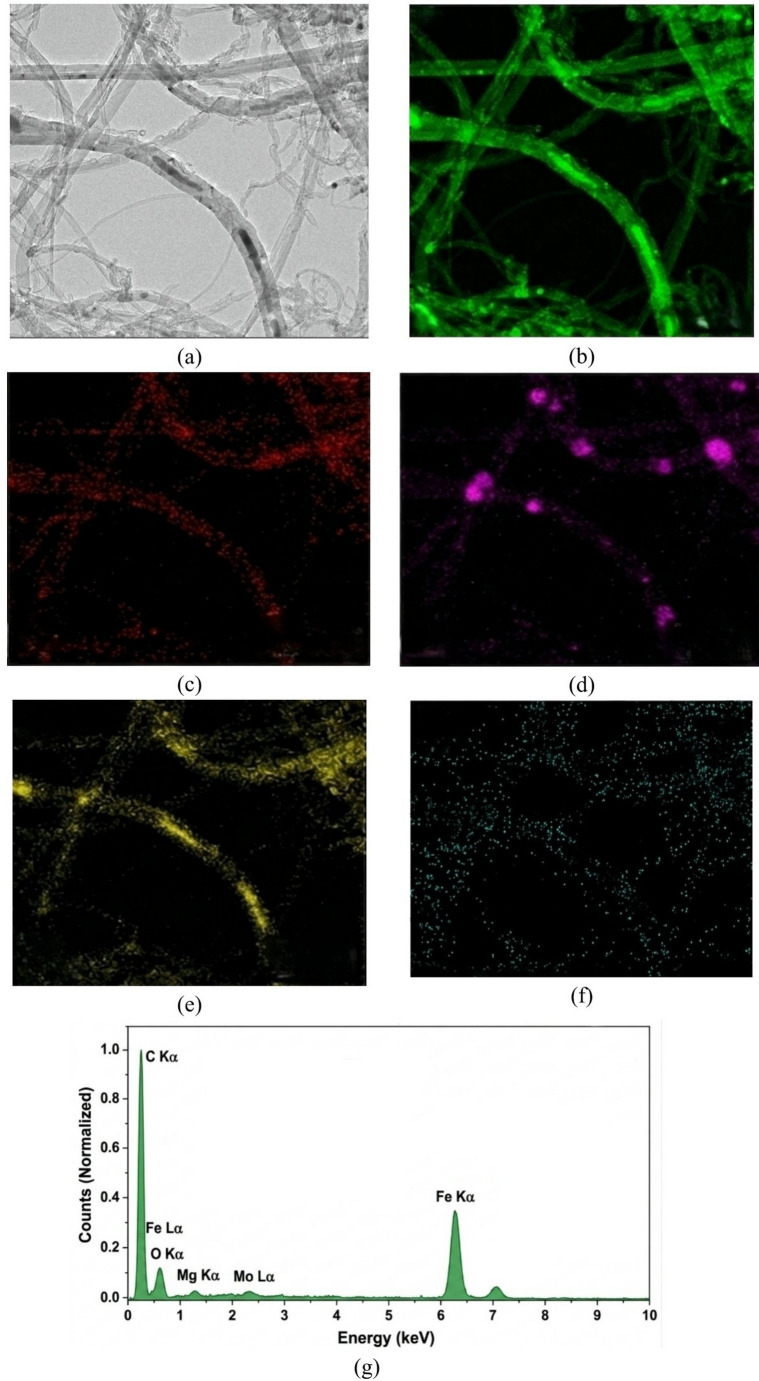
STEM-EDS images of elements, **a**) GNS-CNT, **b**) C, **c**) O, **d**) Fe, **e**) Mo, **f**) Mg, and **g**) EDS.

The analysis focuses on detecting elements in a sample using STEM-EDS maps, highlighting the presence of graphene nanosheet-carbon nanotube hybrids (GNS-CNTs)^80,82^, carbon (C), oxygen (O), iron (Fe), molybdenum (Mo), and magnesium (Mg). The EDX spectrum shows normalized counts versus energy with critical observations at various keV. Magnesium concentration appears to decrease gradually without a distinct peak, which suggests three possibilities: the concentration is very low (below 1–2 wt%), overlapping signals from C/O obscure the peak, or the spectrum is from an area with minimal Mg presence. A low, bright Mg signal indicates the weak concentrations, while sparse dots may align with it, as shown in Fig. 9.

The highest normalized counts were obtained for carbon (C) at 0.5 keV with 0.98, which is a typical characteristic of a high carbon content in carbon nanotubes (CNTs) or graphene nanostructures. Iron (Fe) is a major metallic component with strong normalized counts of 0.35 at 6.5 keV. Its Lα peak at 0.8 keV overlaps with oxygen but is distinguishable. At 0.8 keV, the oxygen (O) signal has low counts (0.05), but it is successfully deconvolved from the iron (Fe) Lα signal. Mg and Mo are detected at 1.0 keV. Mg and Mo are weakly detected but confirmed to be present. Comparison with earlier STEM-EDS maps is in agreement with the EDX results: strong carbon signals, a weaker oxygen map, concentrated bright particles for iron, a weaker molybdenum map co-localized with iron, and a diffuse magnesium presence. In summary, the EDX spectrum indicates high carbon content, moderate iron levels, and low concentrations of magnesium and molybdenum.

### FTIR analysis

**Fig. 10:**
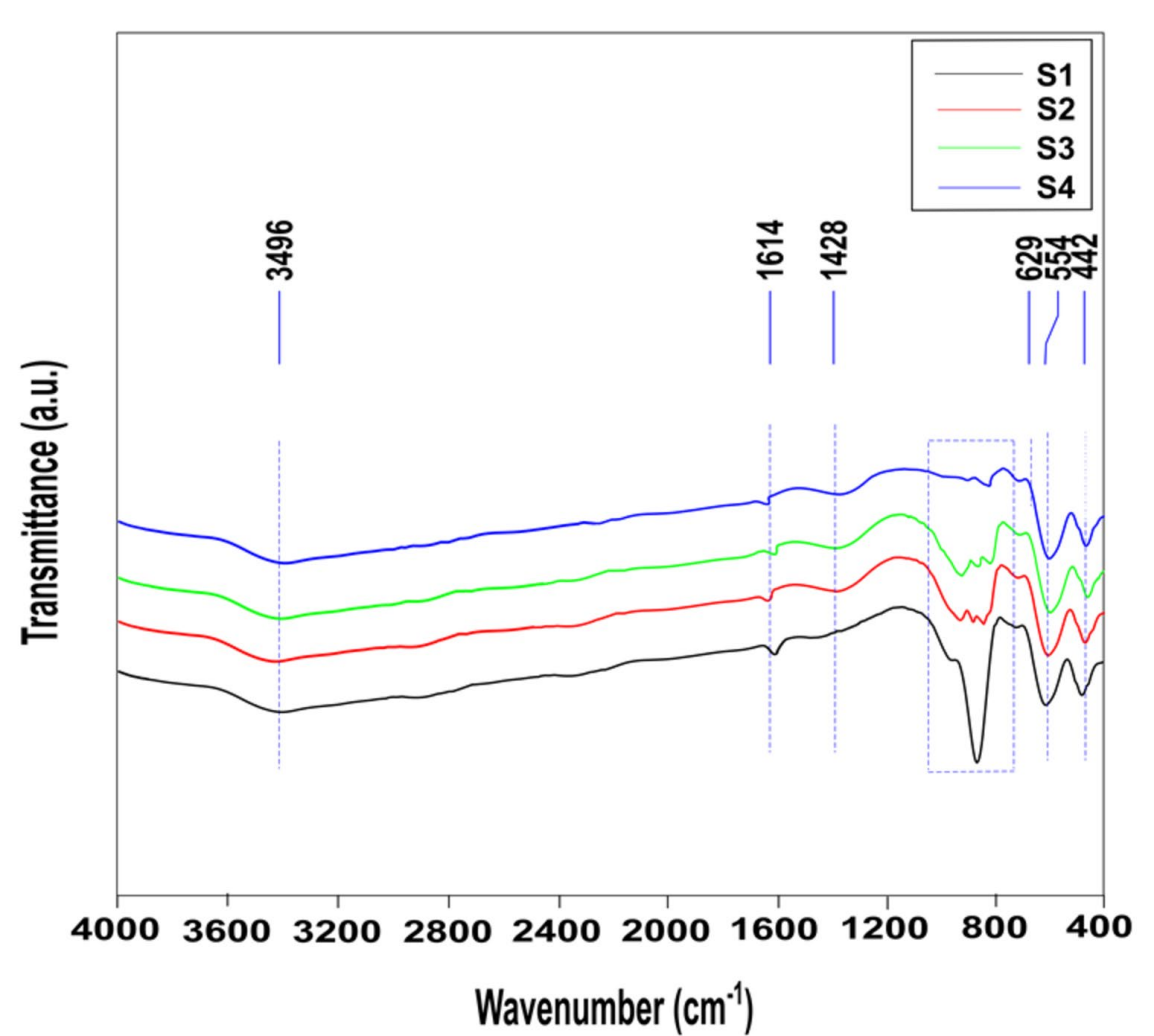
FTIR profiles of Mo-Mg-Fe samples with varying ratios of Mo/Mg: S1, S2, S3, and S4.

Figure 10 shows the FTIR spectra of GNS-GNT nanocomposites containing Mo-Mg-Fe-based catalysts with different Mo/Mg ratios after being heated to 500 °C. The absorption band at 442 cm⁻¹ confirms the presence of MgO in the catalyst, indicating the stretching vibration of the Mg-O bond. The band at 554 cm⁻¹, which is associated with Mo–O and Fe–O stretching in the Fe₂(MoO₄)₃ phase, shows a slight shift toward lower wavenumbers, probably because of its interaction with the nearby oxides of MgO and Fe₂O₃^95^. Additionally, only MgO-rich samples (10Mo–40Mg-Fe) have a shoulder at 629 cm⁻¹, which corresponds to Fe–O vibrations, indicating the presence of free Fe₃O₃ particles on the surface, which disappear at higher Mo content. A broad and intense absorption band appears in the 680–1048 cm⁻¹ range, especially in the S1 sample. This band is mainly associated with Mo–O stretching vibrations from molybdenum in various (Mo⁶⁺, Mo⁵⁺, and Mo⁴⁺) oxidation states^96^. As the amount of MgO increases, this band becomes less intense and starts to break up into several distinct peaks. This shift suggests that MoO₃, MgO, and FeO₃ are interacting to generate mixed oxide phases such as FeMoOₓ and MgMoOₓ. The characteristic peak near 995 cm⁻¹ usually associated with terminal Mo = O bonds is not observed. The production of isolated Mo = O bonds appears to be inhibited by strong chemical interactions between MoO₃ and the MgO/Fe₂O₃ components. S4, S3, and S2 samples exhibit a distinct band at 1428 cm⁻¹. Asymmetric carbonate ion stretching (CO₃²⁻) adsorbed on the MgO surface is linked to this band. A previous study found that MgO’s amphoteric (acid-base) structure makes it effective at capturing CO₂ from the air^97^. The peaks appearing at 1614 cm⁻¹ and 3496 cm⁻¹ are the bending vibrations of water molecules (H-O-H) and stretching of hydroxyl groups (O-H). These peaks indicate that the samples contain adsorbed moisture and surface OH groups, which are typical of hydrated or hydroxylated surfaces. This also confirms the presence of MgO and partially oxidized graphene in the material. In the fingerprint region (1200–1000 cm⁻¹), multiple overlapping peaks also appear, corresponding to C–O stretching vibrations from epoxy, alkoxy, and additional functional groups containing oxygen that are part of the GNS-GNT structure. These characteristics suggest that the graphene-based structure retains certain functional groups or is partially oxidized. Peak intensity differences across the various Mo/Mg ratios indicate that the catalyst’s composition influences both the degree of oxidation and the strength of component interactions.

The graphitic carbon structure was confirmed by Raman spectroscopy, where the D and G bands related to the structural defects and sp^2^ hybridized carbon network were discovered, respectively. XRD analysis confirmed the crystalline carbon and revealed MgO, Mo_2_C and Fe/Fe_3_ C phases. Such crystalline structures containing oxides and carbides are in accordance with metal-oxygen and carbon-associated vibrational bands in FTIR spectra. Energy EDS confirmed the presence of elements C, O, Mg, Mo, and Fe in all samples. The complementing FTIR results confirmed the presence of O-H stretching vibrations at ~ 3496 cm^− 1^ and peaks at ~ 1614 and ~ 1428 cm^− 1^ attributed to the oxygenated functional groups and carbonates in oxidized carbon nanostructures. Additional absorption bands in the 900 –400 cm^− 1^ (629, 554 and 442 cm^− 1^) range were assigned to metal-oxygen vibrations associated with Mg-O, Fe-O, and Mo-O bonds. The FTIR, Raman, XRD, and EDS analysis provided strong evidence for the existence of graphitic carbon, metal oxide-related species, and oxygen-functional groups in the GNS/CNT-MgO-Mo-Fe hybrid system. The Raman quantitative analysis, like ID/IG ratio and band broadening, indicated higher structural disorder and interfacial interactions while the presence of sp^2^-hybridized carbon networks was confirmed by the G band. The findings from FTIR establish a complete understanding of structural characteristics of the material.

### UV-vis analysis

**Fig. 11:**
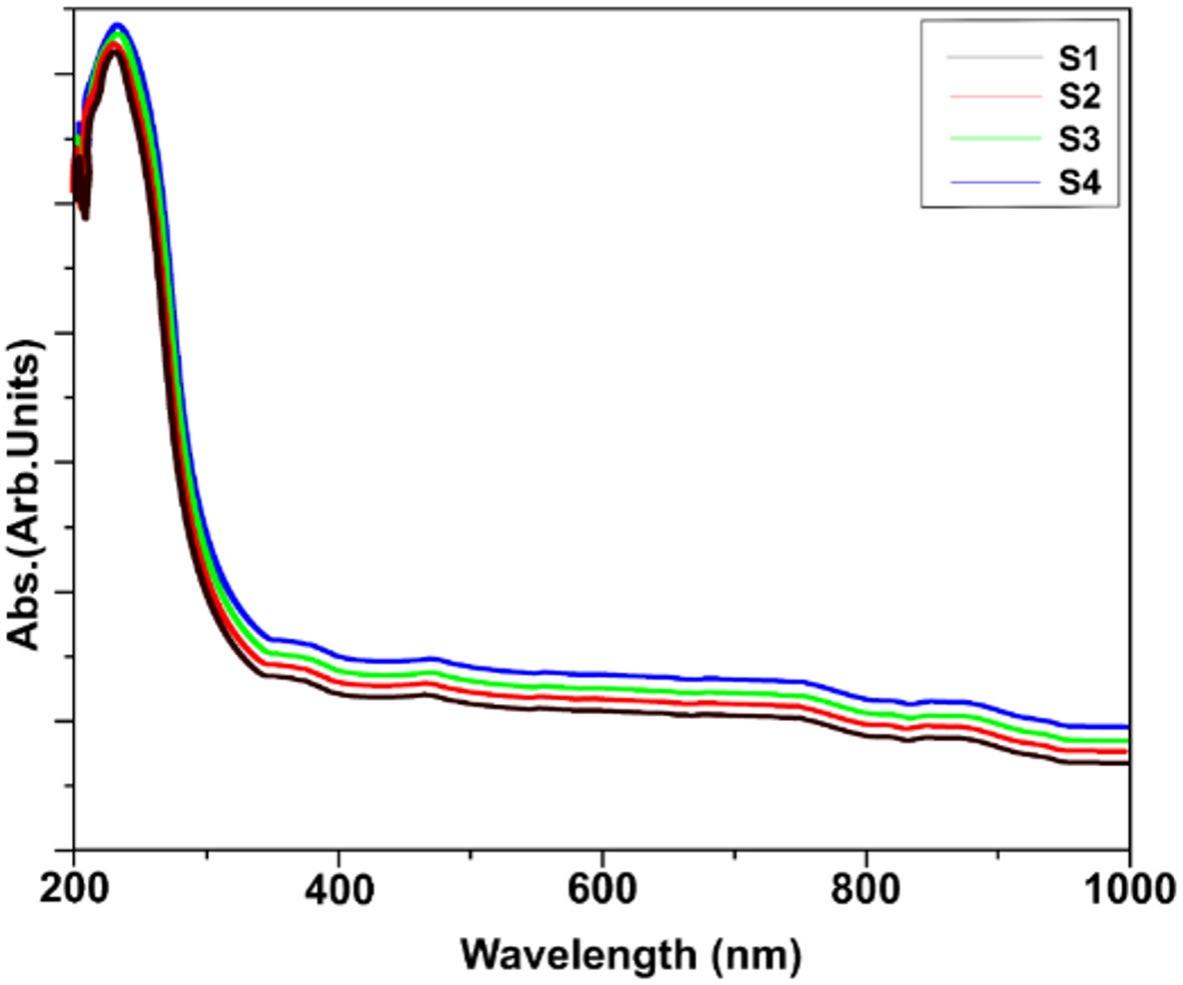
UV–Vis spectra of GNS/CNT hybrid.

The UV-visible absorption spectrum reflects the hybrid GNS-CNT composite’s optical characteristics. As shown in Fig. 11, the spectrum features a broad absorption band ranging from 200 to 300 nm, just below the prominent peak at around 250 nm. After 400 nm, the absorption decreases significantly but doesn’t disappear entirely. A steady absorption tail that is faint persists across the visible and near-infrared (NIR) spectrum, reaching around 1000 nm^98–100^. UV absorption is predominantly induced by π→π* electronic transitions in conjugated C = C bonds, as seen in graphene and carbon nanotubes. The peaks at 267–276 nm are often linked to graphitic domains within graphene nanosheets (GNS) and one-dimensional π-conjugated structures of carbon nanotubes. The combination of graphene and CNTs in the composite increases π-electron delocalization, resulting in strong UV absorption in the spectrum. The gradual decrease in absorbance and the persistent low-level absorption across the visible to NIR region suggest that additional optical processes are taking place within the hybrid material. This pattern indicates the presence of n→π* transitions and surface plasmon resonance effects generated by implanted metal or metal oxide nanoparticles. Hybrid composites, including oxides such as MgO, MoO₃, Fe₂O₃, and others, often show a significant absorbance tail. The UV-Vis spectra after the removal of the catalyst give information on the structure and quality of the GNS-CNT hybrid. The characteristic peaks of the metal-ligand show the presence of metal catalyst such as Fe or Mo. For example, Fe^3+^ complexes can give a peak at about 510 nm. In the Fe-Mo-Mg catalyst system, the components of the catalyst show entirely different absorption mechanisms based on transition metal coordination chemistry and metal-oxide bandgaps. The wide bandgap (~ 7.8 eV) of the magnesium oxide support (Mg-O matrix) band (200–220 nm) means that its fundamental bulk absorption is further into the deep UV. On the other hand, nanocrystalline MgO supports have a sharp band around 210 nm due to excitonic transitions at low-coordination surface sites and the O^2−^ to Mg^2+^ charge transfers. Iron Oxide/Iron-Derived Active Phase (Fe³⁺ Transitions) (~ 250–350 nm and ~ 400–600 nm): The species containing iron generally display two characteristic absorption regions depending on oxidation state, coordination environment and dispersion. The absorption band observed in the 250–350 nm region is commonly assigned to ligand-to-metal charge transfer (LMCT) transitions of oxygen ligands (O^2−^) to Fe^3+^ ions (O^2−^→Fe^3+^) in tetrahedral and octahedral coordination environments. On the other hand, the broad and weaker absorption features in the 400–600 nm range are commonly assigned to spin-forbidden d–d crystal-field transitions of Fe^3+^ ions (⁶A₁g → ⁴T₁g and ⁶ A₁g → ⁴T₂g) in iron oxide clusters or highly dispersed Fe-containing species embedded in the catalyst matrix. Molybdenum species are usually present as Mo ^6+^ in molybdate phases (MgMoO_4_) or as highly dispersed molybdenum oxides and generally do not show d–d electronic transitions because of their d^0^ electronic configuration. Instead, the optical absorption is dominated by intense ligand-to-metal charge-transfer (LMCT) transitions in the near UV. These bands are derived from the transfer of electrons from the oxygen 2p orbitals to the vacant molybdenum 4d orbitals (O²⁻ → Mo⁶⁺) and are usually observed in the wavelength range of 270–320 nm. The observed absorption features and their intensities are indicative of highly oxidized molybdenum species in the catalyst matrix.^101–104^ Finally, the absorption band near 235–300 nm is relatively stable for all samples, suggesting that the basic π-electron structure of the graphitic domains is largely maintained despite changes in the catalyst composition. Such behavior may indicates that the change of the Mo/Mg ratio mainly influences the carbon yield and morphology, without affecting significantly the electronic structure of the graphitic framework. This extended absorption could be due to structural defects, interfacial interactions, and charge-transfer processes between carbon-based components and metal oxides. This broad-based absorption has significant implications. The material’s persistent absorption across the visible and NIR regions, notably with the non-zero tail, indicates that when exposed to high-intensity light, it could undergo nonlinear optical processes such as optical limiting or photothermal conversion.

### Raman analysis

**Fig. 12:**
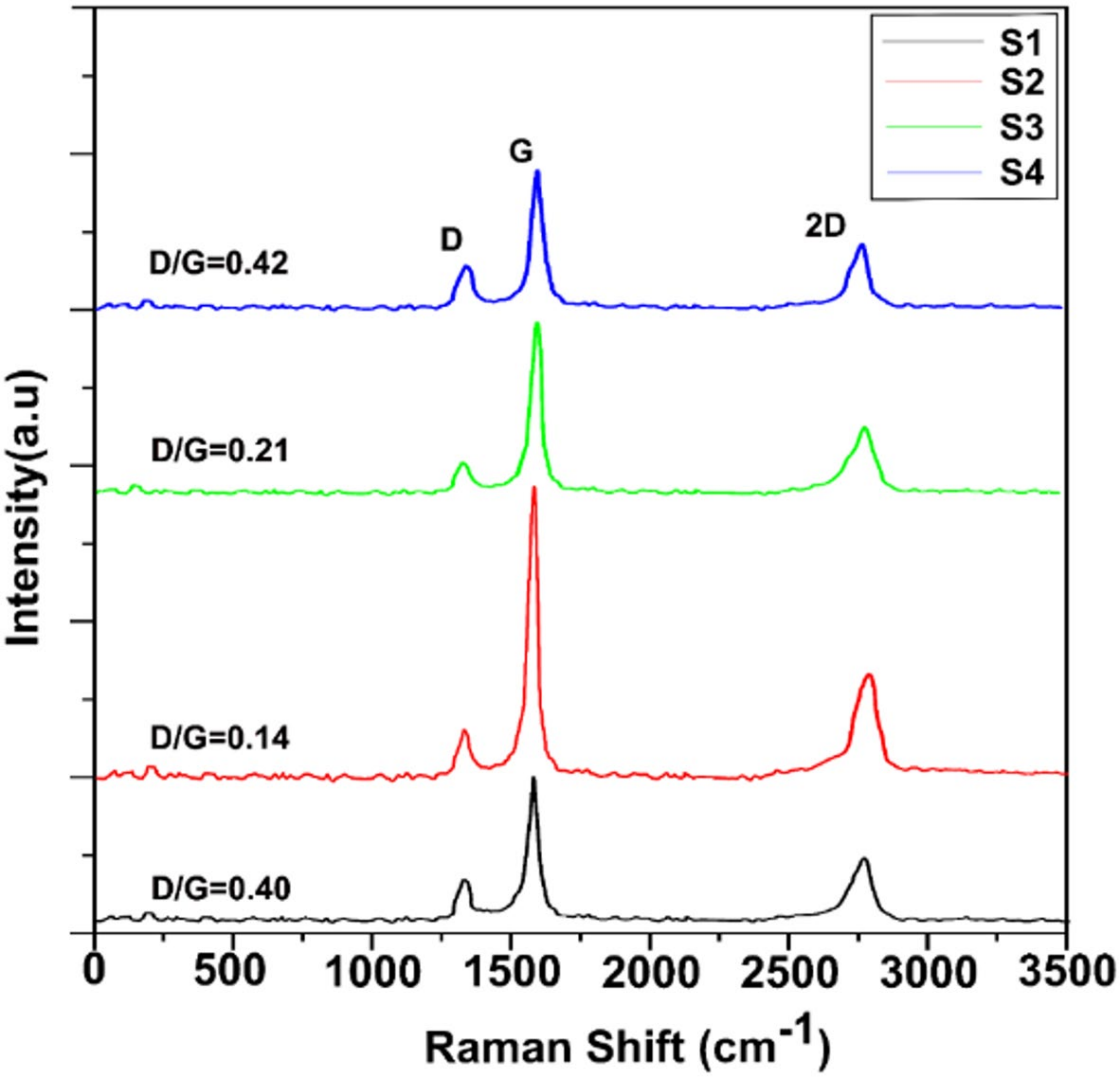
Spent Mo-Mg-Fe catalysts’ Raman spectra with the following Mo/MgO ratios: (**a**) S1, (**b**) S2, (**c**) S3, and (**d**) S4.

The Raman spectra shown correspond to four different hybrid carbon-based samples, likely made up of graphene and nanostructures like carbon nanotubes (CNTs). Each sample displays a different level of structural disorder. The spectra span a Raman shift range from 0 to around 3700 cm⁻¹, with three main peaks clearly visible: the D band (1345 cm⁻¹), the G band (1579 cm⁻¹), and the 2D band (2676 cm⁻¹), as shown in Fig. 12. These peaks are essential for assessing the structural quality of materials made from sp²-bonded carbon atoms. The D band is linked to ring-breathing vibrations in the carbon lattice and only appears when there are defects or disruptions in the structure. Graphitic materials exhibit the G band, which indicates in-plane stretching of sp² carbon atoms^105^. The 2D band, resulting from a two-phonon scattering process, is widely used for the estimation of the graphene layers features (layer number, stacking and degree of graphitic structure)^106^.

The intensity ratio of the D and G bands (I_D_/I_G_) is one of the most essential indicators for assessing the quality of these samples. The D/G ratio determines the degree of disorder or defects in the carbon structure. The top-to-bottom spectra in the figure have D/G ratios of 0.42 (blue), 0.21 (green), 0.14 (red), and 0.40 (black). The blue spectrum with a D/G ratio of 0.42 shows a higher D band than the G band, indicating moderate structural disorder in the sp² carbon structure^38^. The I_D_/I_G_ ratio was calculated from the maximum peak intensities of the D and G bands after baseline correction. Raman spectra were collected from several randomly selected areas on the sample surface (*n* = 5). The obtained ID/IG values showed only minor variations, indicating the homogeneous distribution of the GNS-CNT hybrid and the reproducibility of the Raman results. Raman examination of GNS-CNT samples indicates distinct levels of structural disorder. The defect density and structural quality of GNS-CNT samples can be arranged in a descending order as S2 > S3 > S1 = S4. Sample S2 has the lowest defect density (I_D_/I_G_ = 0.14) and the highest graphitic ordering and is thus the best. S3 (I_D_/I_G_=0.21) demonstrates good graphitic features with a medium level of faults, which indicates mild disorder. S1 (I_D_/I_G_ = 0.40) reveals a large structural disorder due to the larger proportion of faulty carbon sites. Finally, S4 (I_D_/I_G_ = 0.42) has the highest defect density and most structural faults. These observations are confirmed by the area ratio (A_D_/A_G_), which reveals an ordered structure in S2 and more faults in S1 and S4. The study of the Raman spectra reveals that all samples have common characteristics of few-layer to multilayer graphene structures with high ratios I2D/IG = 0.33 and A2D/AG ≈ 0.43. S2 is the sample with the highest graphitic ordering and structural quality among the studied GNS-CNT samples. Important peaks were seen at 1579 cm⁻¹ (G band), 1345 cm⁻¹ (D band), and 2676.1 cm⁻¹ (2D band). Close-up measurements showed: D band (Position: 1344.8 cm⁻¹, FWHM: 42 cm⁻¹, Height: 24.1 a.u., Relative Area: 1012 a.u.), G band (Position: 1579.1 cm⁻¹, FWHM: 50 cm⁻¹, Height: 172 a.u., Relative Area: 8600 a.u.), and 2_D_ band (Position: 2676.1 cm⁻¹, FWHM: 65 cm⁻¹, Height: 57 a.u., Relative Area: 3705 a.u.). The S2 displayed the minimum defect density with an I_D_/I_G_ ratio of 0.14 and an A_D_/A_G_ ratio of ~ 0.14, which reflected the improved graphitic ordering and reduced flaws. Meanwhile, S3 showed moderate disorder (I_D_/I_G_ = 0.21). However, the defect density of S1 and S4 was higher with an I_D_/I_G_ ratio of 0.40 and 0.42, respectively. The computed I_2D_/I_G_ ratios (0.33–0.42) and broad 2D bands (FWHM 65–82 cm⁻¹) show that the GNS-CNT hybrid structure contains few-layer to multilayer graphene sheets, which confirms that S2 is indeed the most structurally superior sample. The 2D band shape, FWHM, and I_2D_/I_G_ ratio quantitatively analyzed in Table 5.

The calculated parameters indicating that a low ID/IG ratio corresponds to fewer defects, an AD/AG ratio indicates a low fraction of defected carbon, and an I₂D/IG ratio suggests a few-layer to multilayer graphene structure. The position of the G band indicates highly graphitized sp² carbon, whereas the position and FWHM of the 2D band suggest few layers of graphene rather than monolayer graphene.

**Table 5:** Complete quantitative Raman analysis with FWHM, 2D band shape, and I_2D_/I_G_ ratio.

Sample	I_D_/I_G_	A_D_/A_G_	I_2D_/I_G_	A₂_D_/A_G_	FWHM(D) (cm⁻¹)	FWHM(G) (cm⁻¹)	FWHM(2D) (cm⁻¹)
S1	0.40	0.43	0.42	0.64	58	54	82
S2	0.14	0.12	0.33	0.43	42	50	65
S3	0.21	0.20	0.37	0.52	48	51	72
S4	0.42	0.46	0.40	0.62	60	55	85

The I₂_D_/I_G_ values (0.33–0.42) together with A₂_D_/A_G_ values below 1 and FWHM (2D) values of 65–85 cm⁻¹ are consistent with few-layer to multilayer graphene nanosheets rather than monolayer graphene. The relationship between the Raman spectrum characteristics and the number of layers as well as the stacking orientation in few-layer graphene is explored in^107^. It demonstrates that the integrated intensity ratio (A₂_D_/A_G_) typically decreases, while the 2D band width (Γ₂_D_) increases with layer number (N) and small rotation angles. FWHM (2D) is a fingerprint of Bernal-stacked few-layer graphene, with a typical range of 52 to 69 cm. This range is larger compared to the values of single-layer graphene^80^. specifications focused on graphene and highlighting the FWHM of the 2D peak as an important parameter to characterize the graphene and to stress its importance in quantitative analysis^81^.

### Thermal analysis

**Fig. 13:**
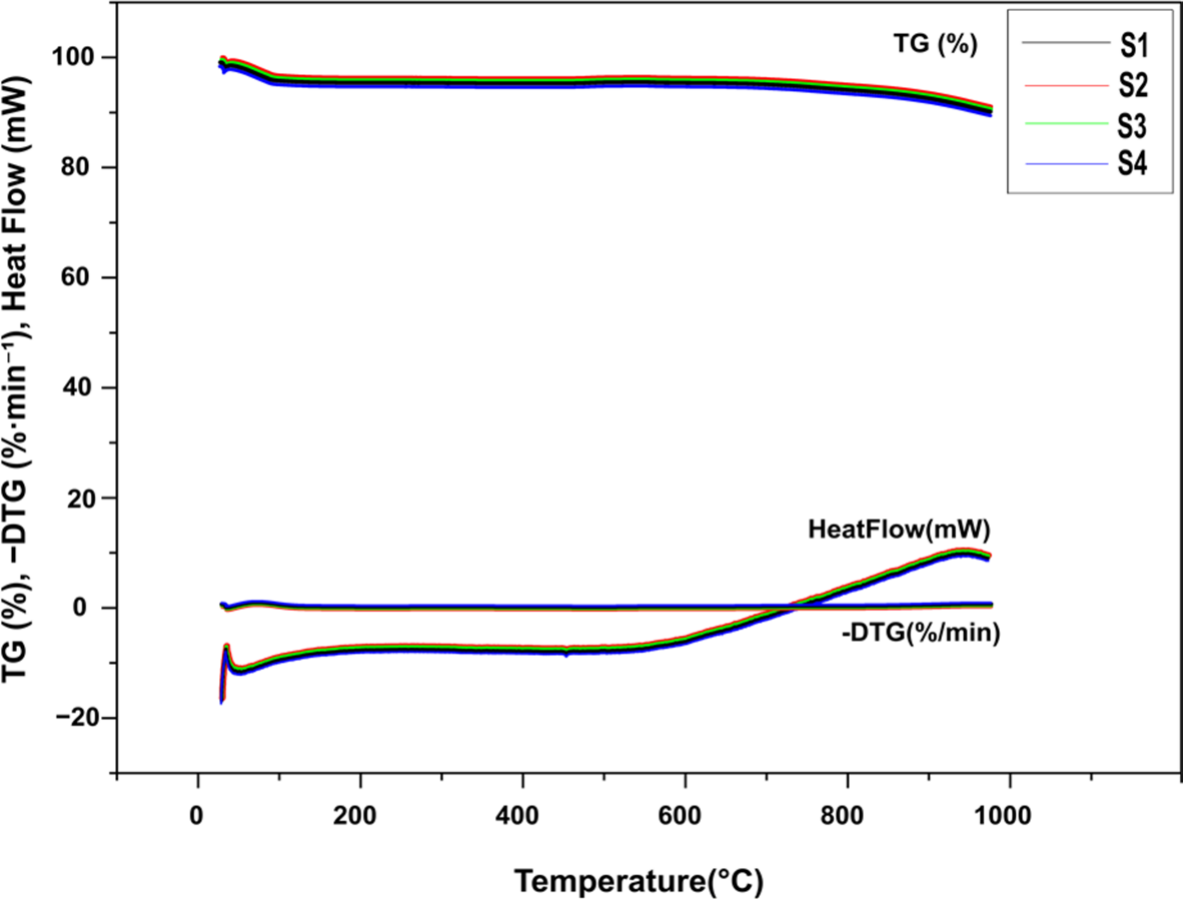
TGA, DTG, and DSC results of GNS-CNT samples in the temperature range from 30 to 1000 °C.

**Fig. 14:**
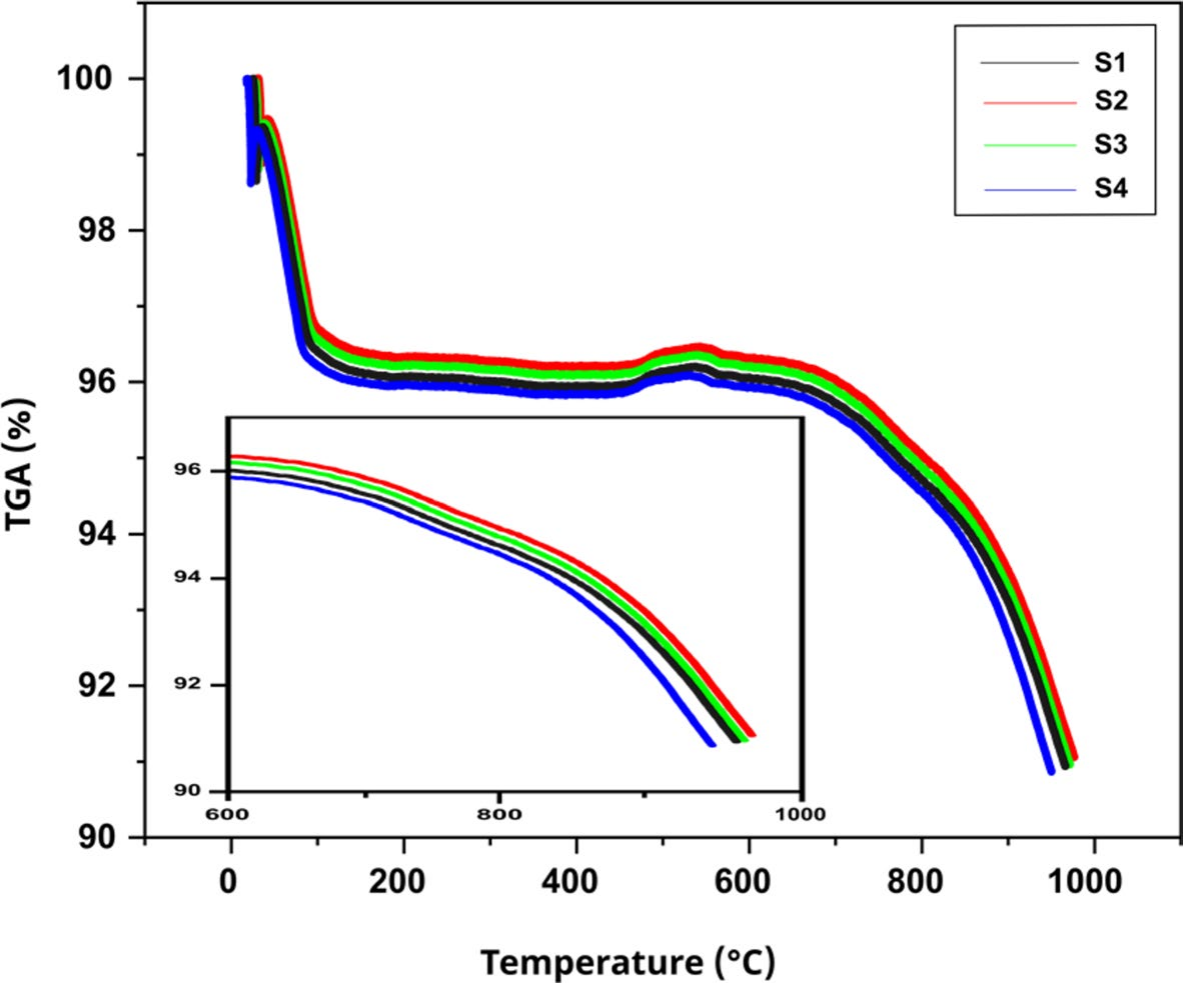
Zoomed view of the TGA curves showing the subtle weight loss behavior between 90–100% mass of GNS-CNT samples.

**Fig. 15:**
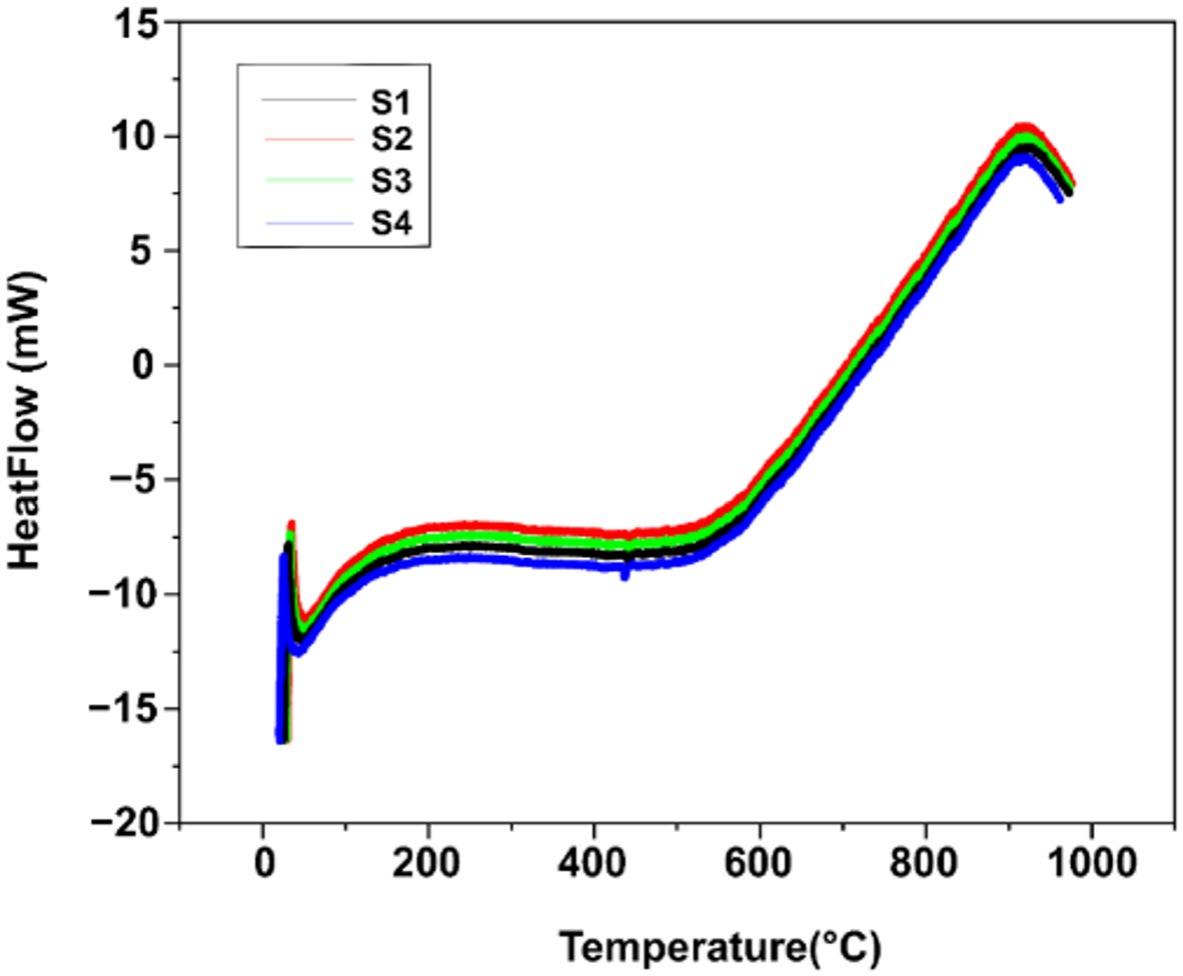
Zoomed view of the DSC curves showing the heat flow behavior of GNS-CNT samples.

The GNS/CNT-Fe-Mo-Mg hybrid system exhibits clear multi-stage thermal behavior, as shown in TGA and DSC figures. An initial small mass loss below 200 °C is related to the removal of adsorbed moisture and volatile surface species, after which the materials remain thermally stable up to 400 °C. The significant exothermic DSC peak at 440–450 °C, accompanied by negligible weight loss, is attributed to Mo₂C production and carbon structural restructuring. This demonstrates DSC’s ability to identify phase alterations that TGA alone cannot^66,97,108^. Thermal measurements of CNT–GNS/Mo–Mg composites conducted under a nitrogen atmosphere at a heating rate of 10 °C min⁻¹ to 1000 °C demonstrate a strong dependence on catalyst composition. Increasing Mg content promotes CNT and overall carbon production, but it also affects thermal stability due to increased Mg volatilization^106,108^. Higher Mo content improves the thermal stability of GNS-CNT hybrid powders by promoting graphitization and forming protective Mo₂C phases. Although all samples have comparable overall TGA patterns (Figs. 13 and 14, and 15), clear differences are observed in the onset decomposition temperatures and total weight loss. Among the studied compositions, sample S2 shows the highest thermal stability, followed by S3, S1, and S4. These variations are primarily quantitative rather than qualitative, indicating that tuning the Mo/Mg ratio effectively adjusts the degree of graphitization and thermal stabilization without altering the fundamental thermal decomposition mechanism of the hybrid system.

At elevated temperatures, the gradual mass loss is primarily due to highly graphitic carbon stabilized by metal carbides and oxides. The Mo/MgO ratio plays a key role in this behavior: the S2 sample (30Mo–20MgO) shows the highest decomposition onset and DTG peak, reflecting enhanced thermal stability compared to MgO-rich compositions, thanks to the protective effect of Mo₂C against oxidation. A clear relationship is observed between Raman defect density and thermal stability: the red sample (I_D_/I_G_ = 0.14) exhibits the greatest oxidation resistance with a T_max_ of 620 °C, followed by the green (580 °C), black (530 °C), and blue (520 °C) samples, corresponding to increasing defect levels and earlier oxidation. Compared with CNT-only materials, which typically oxidize between 400 and 600 °C, and defective graphene at around 500 °C, the GNS-CNT hybrids demonstrate a broader stability range of 520–620 °C. These results indicate that careful catalyst tuning allows precise control over the intrinsic thermal stability of hybrid carbon nanomaterials.

Under inert conditions, the material exhibits enhanced thermal stability, as evidenced by the very limited mass loss of only ~ 2–6% up to 1000 °C. Unlike measurements in oxidative atmospheres, where the weight typically drops sharply from 100% due to carbon combustion, the present weight scale shows only a slight decrease, from about 92–98% down to ~ 90%. This minimal mass change confirms that the carbon framework remains largely intact and that the observed losses arise mainly from the decomposition of surface functional groups, the release of trapped species, and very slow structural reorganization, rather than from bulk carbon degradation or burning. Based on the established thermal stability ranking, the DSC thermograms reveal a systematic progression: in less stable samples (S1, S4), the low-temperature graphitization exotherm (200–400 °C), the catalyst reduction endotherm (550 °C), the alloying/carbide crystallization exotherm (650 °C), and high-temperature phase transitions (800 °C) all occur at progressively lower temperatures. Samples S3 and S2 show clear noticeable shifts toward higher temperatures, indicating improved stability. In contrast, the less stable samples show changes toward lower temperatures, along with broader and stronger low-temperature exothermic features and weaker high-temperature alloying features. These tendencies help explain their higher defect levels and earlier thermal degradation, which are consistent with the Raman and TGA results.

The enhanced thermal stability of the GNS–CNT hybrid material stems from the synergistic structural interaction between graphene nanosheets and carbon nanotubes. Scanning electron microscopy (SEM) and transmission electron microscopy (TEM) investigations reveal a close integration of the graphene sheets within the tubular carbon nanotube (CNT) network. This results in a continuous, three-dimensional carbon structure. This interconnected structure improves structural integrity and reduces the density of defect sites that can act as initiation points for thermal degradation. Raman spectroscopy corroborates the substantial graphitic ordering within the hybrid material, as evidenced by the comparatively low I_D_/I_G_ ratio = 0.14. The TGA data supports the idea that this improved graphitic structure helps resist thermal decomposition. Therefore, the combination of structural and graphitic features in the GNS–CNT hybrid leads to better thermal stability. This characteristic is advantageous for applications demanding high thermal resistance, including photonic devices, thermal management systems, and high-power laser protection.

## Conclusion

This study presents a controlled, one-step method for producing GNS/CNT hybrid nanocomposites using methane chemical vapor deposition (CVD). Their properties depend on the Mo/Mg ratio. XRD and TPR results showed that the active phases of Fe-Mo-Mg catalysts were mainly iron molybdate and magnesioferrite species. The results show a clear link between structure, composition, and properties. Specifically, systems with a high Mo content promote the ordering of graphitic layers, improve the stabilization of carbon through carbide formation, and significantly increase resistance to high temperatures by reducing the loss of Mg. The growth of hollow carbon nanotubes (CNTs) is facilitated by systems rich in magnesium oxide (MgO). This is due to the improved distribution of the catalyst and the formation of a mixed-oxide phase. Comprehensive structural and spectroscopic investigations validate the presence of low defect densities, enhanced elemental distribution, and well-integrated hybrid interfaces. SAED, TEM, and SEM exhibited the coexistence of graphene sheets attached with CNTs. EDS and STEM indicated the enhanced distribution of C, O, Mg, Mo, and Fe. Raman showed low flaws (ID/IG ≈ 0.14), I2D/IG = 0.33 and A2D/AG ≈ 0.43 indicating that the samples had common properties of few to multilayer graphene structures. Fourier-transform infrared spectroscopy (FTIR) reflected Mo/Mg dependent oxide and surface chemistry, whereas UV–Vis spectroscopy showed broad, increased optical absorption. These materials are distinguished from typical oxidation-based graphene derivatives by their enhanced thermal stability in an inert atmosphere, characterized by a mass loss of no more than 6% up to 1000 °C, thereby underscoring the efficacy of carbide-assisted carbon protection mechanisms. The research highlights the Mo/Mg ratio’s role in controlling defect density, oxide chemistry, graphitic structure, and optical absorption in hybrid carbon frameworks. This understanding offers a way to design carbon nanohybrids that are thermally stable, optically active, and structurally sound. The CVD-derived GNS/CNT platform developed here is a versatile materials system. It shows considerable potential for high-performance applications in advanced electronics, photonics, catalysis, nonlinear optics, and technologies designed for extreme environments. The prepared hybrid composites are important candidates for their superior optical and thermal properties. The current work under investigation is studying the nonlinear optical properties of the synthesized hybrids in polymeric hosts via the z-scan technique across multiple wavelengths, enabling the identification of the optimal hybrid-polymer configuration for high-efficiency optical limiting and photonic device integration. (355, 532, and 1064 nm) will allow precise correlation between composite composition, polymer environment, and NLO performance.

## Data Availability

Data will be made available on request.
